# Testing and tracking in the UK: A dynamic causal modelling study

**DOI:** 10.12688/wellcomeopenres.16004.1

**Published:** 2020-06-17

**Authors:** Karl J. Friston, Thomas Parr, Peter Zeidman, Adeel Razi, Guillaume Flandin, Jean Daunizeau, Oliver J. Hulme, Alexander J. Billig, Vladimir Litvak, Cathy J. Price, Rosalyn J. Moran, Christian Lambert

**Affiliations:** 1 The Wellcome Centre for Human Neuroimaging, University College London, London, WC1N 3BG, UK; 2 Turner Institute for Brain and Mental Health, Monash University, Clayton, VIC, 3800, Australia; 3 Institut du Cerveau et de la Moelle épinière, INSERM UMRS, Paris, France; 4 Danish Research Centre for Magnetic Resonance, Centre for Functional and Diagnostic Imaging and Research, Copenhagen University Hospital Hvidovre, Hvidovre, Denmark; 5 London Mathematical Laboratory, London, UK; 6 Ear Institute, University College London, London, UK; 7 Centre for Neuroimaging Science, Department of Neuroimaging, IoPPN, King's College London SE1 1UL, London, UK

**Keywords:** coronavirus, epidemiology, compartmental models, dynamic causal modelling, variational, Bayesian

## Abstract

By equipping a previously reported dynamic causal modelling of COVID-19 with an isolation state, we were able to model the effects of self-isolation consequent on testing and tracking. Specifically, we included a quarantine or isolation state occupied by people who believe they might be infected but are asymptomatic—and could only leave if they test negative. We recovered *maximum posteriori* estimates of the model parameters using time series of new cases, daily deaths, and tests for the UK. These parameters were used to simulate the trajectory of the outbreak in the UK over an 18-month period. Several clear-cut conclusions emerged from these simulations. For example, under plausible (graded) relaxations of social distancing, a rebound of infections is highly unlikely. The emergence of a second wave depends almost exclusively on the rate at which we lose immunity, inherited from the first wave. There exists no testing strategy that can attenuate mortality rates, other than by deferring or delaying a second wave. A testing and tracking policy—implemented at the present time—will defer any second wave beyond a time horizon of 18 months. Crucially, this deferment is within current testing capabilities (requiring an efficacy of tracing and tracking of about 20% of asymptomatic infected cases, with 50,000 tests per day). These conclusions are based upon a dynamic causal model for which we provide some construct and face validation—using a comparative analysis of the United Kingdom and Germany, supplemented with recent serological studies.

## Introduction

This is the third in a series of technical reports that use dynamic causal modelling to explain and predict the current outbreak of COVID-19. The first report described an enhanced compartmental model based upon a factorisation of latent or hidden states generating timeseries data, such as new cases and daily deaths ( Friston *et al.*, 2020a). This model was concerned with an outbreak in a single region parameterised with an effective population size. The second report assembled several models of a single region, coupled by population flux between regions, to model the pandemic in the united states of America ( Friston *et al.*, 2020b). The focus of this multi-region model was on the genesis of second waves and a key, mechanistic, distinction between rebounds due to premature relaxation of social distancing and second waves due to loss of immunity. The basic conclusions of this second report were that a devolved social distancing strategy—that was sensitive to local metrics—predicted better outcomes than a national or federal strategy. In this report, we return to the model of a single region or country and look more closely at strategies in terms of surveillance; specifically, the role of testing, tracing, and tracking.

The efficacy of contact tracing programs are now the focus of several modelling initiatives ( Aleta *et al.*, 2020; Ferretti *et al.*, 2020; Giordano *et al.*, 2020; Gurdasani & Ziauddeen, 2020; Hellewell *et al.*, 2020; Keeling *et al.*, 2020), whose conclusions depend upon the form of the models used. Models that include social distancing and isolation of infected contacts suggest that a ‘find’, ‘track’, ‘trace’ and ‘isolate’ (FTTI) policy can ameliorate morbidity ( Giordano *et al.*, 2020; Kretzschmar *et al.*, 2020).

To address the efficacy of FTTI, we equipped the dynamic causal model (DCM) with a further location state; namely, a state of self-isolation or quarantine. People entered the state when experiencing symptoms or awaiting a PCR test. They remained isolated for seven days unless they received news that the test was negative. This construction accommodates the mechanistic process by which FTTI operates. In other words, the agenda behind testing and tracking is to isolate those people who are infected before they become contagious. This allows one to move back in time and pre-empt the reproduction of the virus in the population. However, to do this, it is necessary to identify people who are asymptomatic, thereby enriching or enhancing the probability that targeted testing will identify infected individuals. We operationalise this testing and tracking strategy in terms of its efficacy. Here, efficacy is defined as the probability that I will be offered a test by a programme of test and tracking if I am infected and asymptomatic. Under this parameterisation, an ineffective testing and tracking renders this probability zero. Conversely, and efficiency of 100% means that if I am infected and asymptomatic, I will certainly be tested. Clearly, for a large population, high levels of efficacy may not be attainable; however, lower levels may be sufficient to either suppress the reproduction rate of viral transmission ( Aleta *et al.*, 2020) or defer the emergence of any second wave until an efficacious programme of vaccination is in place (or effective treatments have been established).

To model different aspects of testing and surveillance, we had to carefully parameterise testing along a number of dimensions. To do this, we assume that there was a small, time-dependent probability of being tested on any given day. This *testing* probability was modelled in terms of a constant *baseline*, a testing component *sensitive* to the prevalence of infection in the population and a *sustained* component following the first wave. This sustained component was modelled as proportional to the level of herd immunity acquired after successive waves of infection. Having parameterised the testing probability, the *selectivity* of testing was parameterised in terms of the probability of being tested if infected, relative to not being infected. Finally, if I am infected but asymptomatic, then the probability of being tested is supplemented with a *test and track* component—that could start at the beginning of the outbreak, or any subsequent time. This may sound a rather involved parameterisation; however, it is a minimal model needed to generate the number of positive cases reported, given the latent prevalence of infection. This follows because the number of positive cases depends not only on the probability of being tested but whether I am more likely to be tested if I am infected (e.g., I work in a care home) or not (e.g., I have been selected at random by a screening survey).

**Figure 1.**
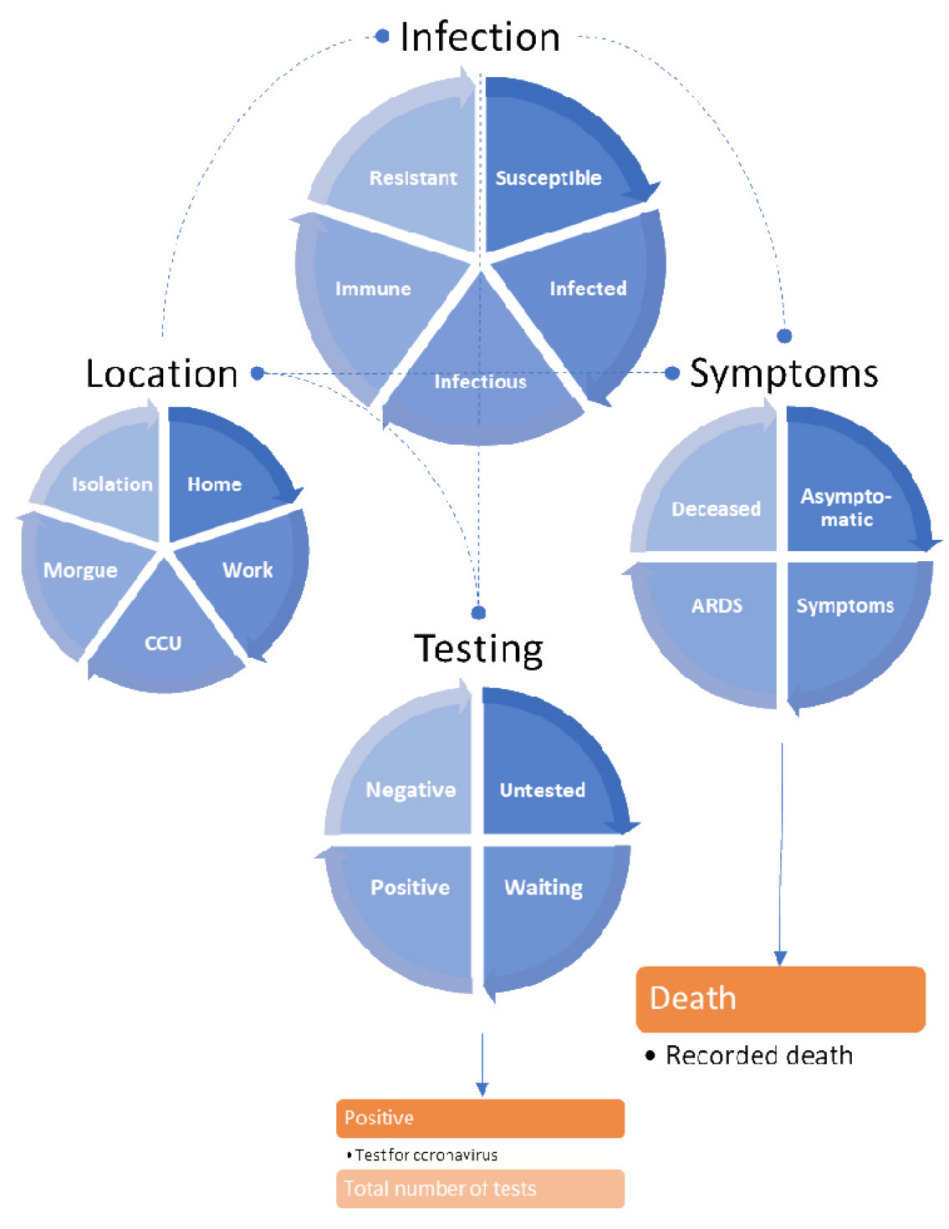
The LIST model. This schematic summarises the LIST ( *location*, *infection symptom* and *testing*) generative model used for the following simulations. This model is formally similar to that described in ( Friston *et al.*, 2020b). Here, it has been augmented with an extra location state ( *isolation*) to model people who are self-isolating because they think they may be infectious. Note that in this model there are no absorbing states. In other words, one can leave any state within any of the four factors. For example, one only occupies the state of being deceased (or testing positive and negative) for a day and then moves to asymptomatic (or untested) on the following day. This ensures that the total population is conserved, i.e., probability mass is conserved in terms of the ensemble density. Furthermore, it enables the occupancy of various states to be interpreted in terms of the rate of daily expression. The blue boxes correspond to states or compartments. The states within any factor are mutually exclusive, where the factors embody the factorial form of this compartmental model. In other words, every individual in the population has to be in one of several possible states that are characterised in terms of four factors or attributes. The orange boxes represent the outputs that are generated by this dynamic causal model, in this instance, daily reports of positive tests, daily tests and deaths.

Please see Figure 1 and Table 1 for a brief review of the model (and the Methods for the parameterisation of self-isolation and testing). With this model and its parameters in place, one can now fit the model to empirical data until the present day. Crucially, because the parameters of this model do not change, they can then be used to forecast the future trajectory, under various adjustments to the testing parameters. The following conclusions foreshadow the results of these simulations:

- There is no plausible parameterisation of the model that would or could permit a flareup or rebound of the outbreak following a relaxation of social distancing measures. This is under the qualified assumption that social distancing continues to be operating in the way it is modelled—and inferred—on the basis of the empirical evidence to date. In short, provided there is a graded, parametric response to the prevalence of infection in the population, there will be no rebound in the weeks following the peak of the first wave.
- A second wave is inevitable. The timing of the second wave depends almost exclusively on the rate at which immunity is lost. In other words, under the assumption that being infected confers immunity—and that the immunity lasts for a given period—the period of immunity determines the timing of the second wave. This second wave is mechanistically distinct from a fluctuation of, or rebound from, the first wave.
- There is no social distancing or surveillance strategy that will have any material impact on the total number of deaths accumulated from the onset of an outbreak to an idealised endemic equilibrium. However, certain strategies can defer waves of infection. Specifically, testing and tracking can defer expression of the second wave beyond a time horizon, after which vaccination or other therapeutic interventions will render it innocuous. In short, the mechanism by which strategic interventions operate is not by eliminating the infection but by slowing it down sufficiently, so that its pathogenicity is dissolved by viral mutation, vaccination, or therapeutic advances. Here, we assume a time horizon of 18 months.
- The most efficacious strategy for deferring a second wave is testing and tracking. Furthermore, the logistic requirements are within current capabilities. The same is not true of the first wave. In other words, it would not have been possible to suppress the first wave with testing and tracking because one would have had to have identified nearly every infected, asymptomatic person in the country and this would have required about over a million tests a week.
- Although, in principle, it is mathematically possible to defer the first wave, one would require either a very small population or a very large testing capacity. Furthermore, the efficacy of testing and tracking would have to be implausibly high, i.e., around 80%.
- The differences between the United Kingdom and Germany are eminently explainable under a dynamic causal model. As might have been anticipated, Germany has a greater propensity to test; however, this testing is substantially less selective for infected individuals than in the UK. Furthermore, the component due to testing and tracking during the first wave was *less* evident than the United Kingdom. This means that pressure is put on other parameters to explain the remarkably low fatality rates in Germany. It appears that—or it looks as if—Germany has a population whose host factors render it more resistant to infection. Furthermore, the fatality rates in critical care are substantially lower in Germany. In short, the explanation for the reduced fatalities in Germany probably lies in clinical surveillance and management, not on their testing and tracking.

**Table 1.** Parameters of the epidemic (LIST) model and priors, *N*( *η*, *C*) (NB: prior means are for scale parameters *θ* = exp( *ϑ*)).

Number	Parameter	Mean	Variance	Description
**1**	*θ _n_*	4	1	Number of initial cases
**2**	*θ _r_*	1/3	1/16	Proportion of resistant cases
**3**	*θ _N_*	66	0	Population size (millions)
**Location**				
**4**	*θ _out_*	1/3	1/256	Probability of going out
**5**	*θ _sde_*	1/32	1/256	Social distancing threshold
**6**	*θ _cap_*	16/100000	1/16	CCU capacity threshold (per capita)
**Infection**				
**7**	*θ _Rin_*	4	1/256	Effective number of contacts: home
**8**	*θ _Rou_*	48	1/256	Effective number of contacts: work
**9**	*θ _trn_*	1/3	1/256	Transmission strength
**10**	$\theta_{inf}=\text{exp}\left(-\frac{1}{\tau_{inf}}\right)$	*τ _inf_* = 4	1/16	Infected period (days)
**11**	$\theta_{con}=\text{exp}\left(-\frac{1}{\tau_{con}}\right)$	*τ _con_* = 4	1/16	Infectious period (days)
**12**	$\theta_{imm}=\text{exp}\left(-\frac{1}{\tau_{imm}}\right)$	*τ _imm_* = 16	0	Period of immunity (months)
**Symptoms**				
**13**	$1-\theta_{dev}=\exp\left(-\frac{1}{\tau_{inc}}\right)$	*τ _inc_* = 5	1/256	Incubation period (days)
**14**	*θ _sev_*	1/128	1/256	Probability of ARDS
**15**	$\theta_{sym}=\text{exp}\left(-\frac{1}{\tau_{sym}}\right)$	*τ _sym_* = 8	1/256	Symptomatic period (days)
**16**	$\theta_{rds}=\text{exp}\left(-\frac{1}{\tau_{rds}}\right)$	*τ _rds_* = 10	1/256	ARDS period (days)
**17**	*θ _fat_*	1/3	1/256	ARDS fatality rate: CCU
**18**	*θ _sur_*	1/8	1/256	ARDS fatality rate: home
**Testing**				
**19**	*θ _ttt_*	1/10000	1	Efficacy of testing and tracking
**20**	*θ _sen_*	1/10000	1	Sensitivity of testing
**21**	*θ _exp_*	1/10000	1	Sustained testing
**22**	*θ _bas_*	8/10000	1	Baseline testing
**23**	*θ _tes_*	1	1	Selectivity of testing
**24**	$\theta_{del}=\text{exp}\left(-\frac{1}{\tau_{del}}\right)$	*τ _del_* = 2	1/4	Delay in reporting test results (days)

In what follows, we will look at the results of simulations that license the above conclusions and unpack these conclusions quantitatively, with a special emphasis on the mechanisms and processes leading to different outcomes.

**Secondary sources** ( Huang *et al.*, 2020; Kissler *et al.*, 2020; Mizumoto & Chowell, 2020; Russell *et al.*, 2020; Verity *et al.*, 2020; Wang *et al.*, 2020) and:

- https://www.statista.com/chart/21105/number-of-critical-care-beds-per-100000-inhabitants/
- https://www.gov.uk/guidance/coronavirus-COVID-19-information-for-the-public
- http://www.imperial.ac.uk/mrc-global-infectious-disease-analysis/COVID-19/

These prior expectations should be read as the effective rates and time constants as they manifest in a real-world setting. For example, a four-day period of contagion is shorter than the period that someone might be infectious ( Wölfel *et al.*, 2020) ^1^, on the (prior) assumption that they will self-isolate, when they realise they could be contagious. Although the scale parameters are implemented as probabilities or rates, they are estimated as log parameters, denoted by *ϑ* = ln *θ*.

### Dynamic causal modelling

Figure 1 provides a schematic that summarises the dynamic causal model used for subsequent inference and simulations. This model can be regarded as a factorial extension of conventional (compartmental) epidemiological models ( Friston *et al.*, 2020a). The factorial aspect means that there are several attributes in play when trying to model the causes of mortality and morbidity. Specifically, it considers the location, infection status, symptomatology, and testing status of any individual in a population. This means that for each factor, there is a certain probability of finding someone in a particular state. Movement from one state to another is parameterised in terms of transition probabilities or rate constants. For example, the probability that I will stay in a state of being *infectious* can be parameterised in terms of the expected number of days that I am contagious. Crucially, the transitions among states within each factor depend upon the states of other factors. These dependencies are denoted by the dashed lines. For example, the probability that I will move from a state of having no symptoms ( *asymptomatic*) to symptoms depends upon whether I am *infected* or not. Note that separating the latent or hidden causes of observable data in this way means that it is possible to be infected but have no symptoms – and *vice versa*. This model is formally the same as previous models ( Friston *et al.*, 2020b); however, we have introduced a fifth location state called *isolation*. This state is entered whenever I have *symptoms* or am waiting to find out whether I test *positive*. The key mechanism—that compels me to enter *isolation*—is a testing and tracking (FTTI) program that alerted me to the possibility of being infected prior to developing symptoms. In this quarantined isolation, I will remain for a given period (seven days), unless I receive notice that I have tested negative following a PCR test. Please see the Methods for a formal parameterisation of these contingencies and how testing data are generated.

**Figure 2.**
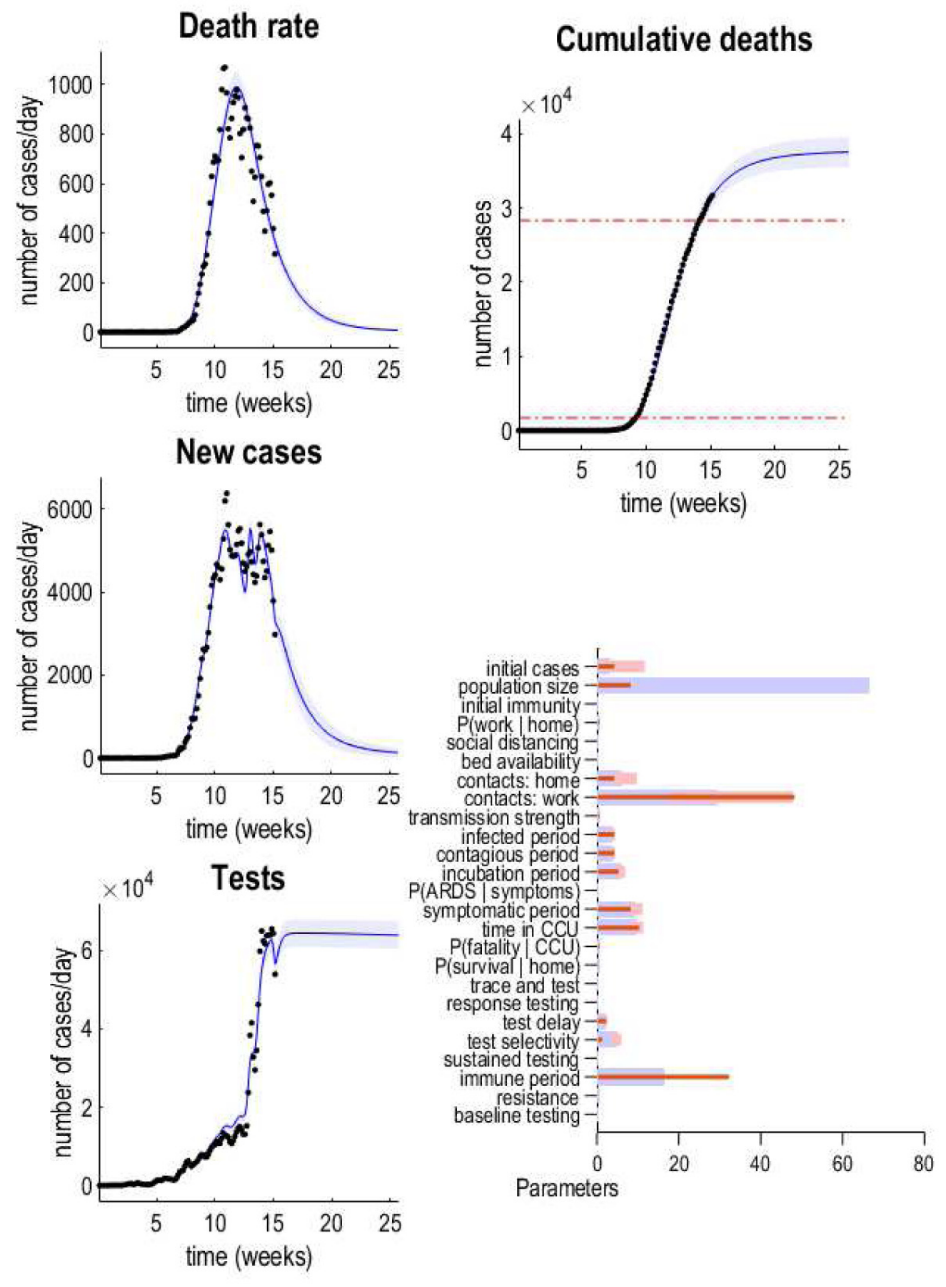
Posterior predictive densities. The panels on the left show the posterior predictive densities over some key outcomes. Here, daily death rates, new positive cases and tests performed. The blue lines represent the posterior expectation and the shaded areas the 90% Bayesian credible intervals. The black dots corresponds to empirical data used to fit the model and estimate posteriors over the model parameters (i.e., the transition probabilities or rate constants in Table 1). The lower right panel reports the posterior parameter densities in terms of their posterior expectation (blue bars) and 90% credible intervals (pink bars). The red bars correspond to the prior expectations. Please see Table 1 for complete specification of the prior densities. The upper right panel shows the cumulative deaths expected under these parameters. The two dashed lines are for reference and correspond to yearly mortality rates for seasonal influenza (from 2014/15 and 2018) ^4^.

With this model in place, one can use standard variational procedures to fit any data at hand ( Friston *et al.*, 2007). Here, we used the daily reports of new (positive) cases and deaths from Johns Hopkins University ^2^ and supplemented this with data from the UK on the total number of tests performed ^3^. The inversion of this model takes about a minute on a personal computer, enabling one to generate posterior estimates of the parameters and accompanying trajectories of hidden states. Figure 2 shows the results of this kind of analysis for timeseries data at the point of writing (10th May 2020). The left panels show the data (dots) superimposed upon a posterior predictive density. This density is a probabilistic statement about the most likely outcomes under the model. Here, it is summarised in terms of the posterior expectation or most likely outcome and 90% Bayesian credible intervals (blue lines and shaded areas, respectively). The parameters upon which these predictions are based are shown in the lower right panel. The outcomes can either be expressed in terms of daily rates or cumulative outcomes over time; for example, the cumulative deaths over a six-month period (as shown on the upper right panel). Notice that these posterior predictive densities cover the past and the future. In other words, they are generated from parameter estimates that do not change in time. This means that one can regard these results as a best fit to the observed data to date. Alternatively, they can be regarded as a forecast of the future. These results suggest that we are nearly halfway down the decline in daily death rates, following the peak (in early April). Crucially, because we have a generative model underneath these data, we can also generate data that has not yet been observed.

**Figure 3.**
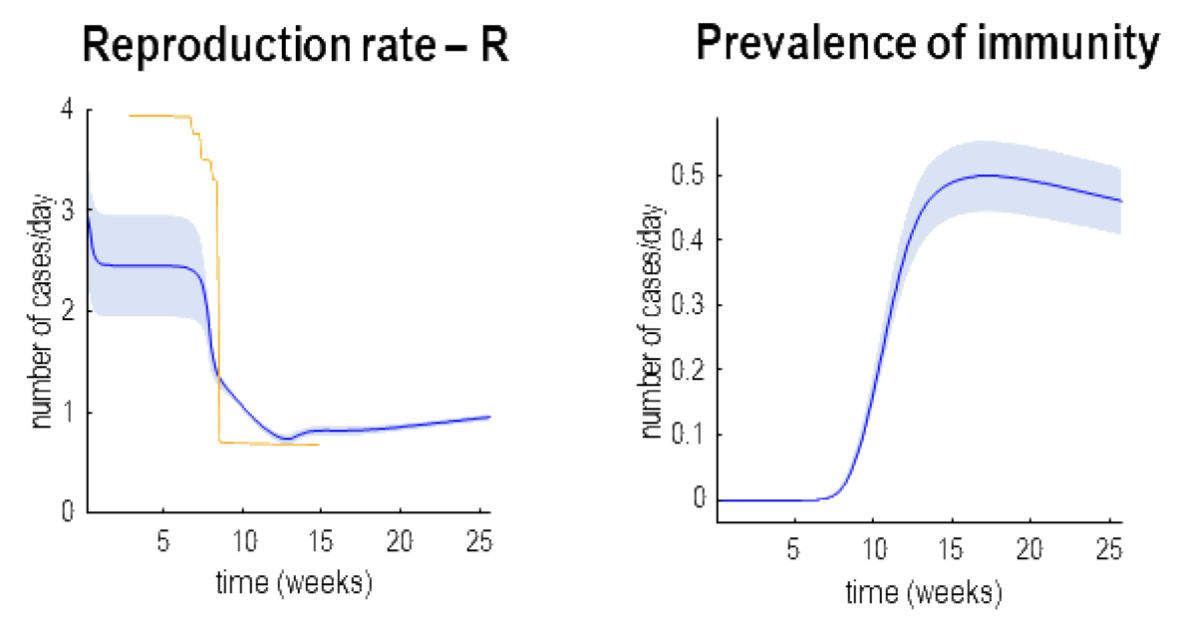
Reproduction rates and herd community. This figure supplements the previous figure with posterior estimates of the reproduction rate (please see Methods) and the prevalence of immunity (a.k.a. herd immunity). Again, the lines represent posterior expectations and the shaded areas 90% credible intervals. The yellow line in the right panel depicts estimates based upon a Bayesian regression model. These are the kind of estimates used to inform government policy. Please see main text for further discussion.

Figure 3 provides two examples of this, in terms of the effective reproduction rate (R) and the prevalence of immunity in the left and right panels, respectively. The prevalence of immunity (a.k.a. herd immunity) is interesting because it is potentially measurable, if we had serological testing of sufficient sensitivity and specificity ( Winter & Hegde, 2020; Yong *et al.*, 2020). When these data become available, they then can be used to improve the posterior estimates of the parameters and shrink uncertainty about the trajectory of seroprevalence ( Vespignani *et al.*, 2020) or immunity ( Kissler *et al.*, 2020).

Notice that the reproduction rate is treated as an outcome. This is an important conceptual point. The reproduction rate is not a cause of fatality—it is a measure or consequence of the underlying causes. These can be computed from the changes in the prevalence of infection and the expected duration of being contagious (please see Methods). Here, the reproduction rate starts at just under three and then falls quickly at the onset of social distancing to about 0.6. In the future, it will gently rise as herd immunity is lost and may ultimately foreshadow a second wave (see below). The yellow line corresponds to the best available estimates of the reproduction ratio based on hierarchical Bayesian Regression, using specific known covariates (regressors) corresponding to different stages of lockdown ( Flaxman *et al.*, 2020).

These estimates ^5^ are the kind of numbers used to currently to guide governmental policy in the UK. They can be regarded as the best estimates from state-of-the-art curve fitting. The key observation here is that they necessarily depend upon data that have already been observed. In other words, they summarise the recent past. This can be seen by comparing the yellow line with the blue line in Figure 3. The real-time estimates afforded by dynamic causal modelling (blue line) evince a more nuanced decline that precedes the sharp drop in conventional estimators (yellow line). This speaks to the potential advantage of using estimates of late states to furnish real-time or instantaneous estimates of the reproduction rate (as opposed to curve fitting or Bayesian regression estimators).

**Figure 4.**
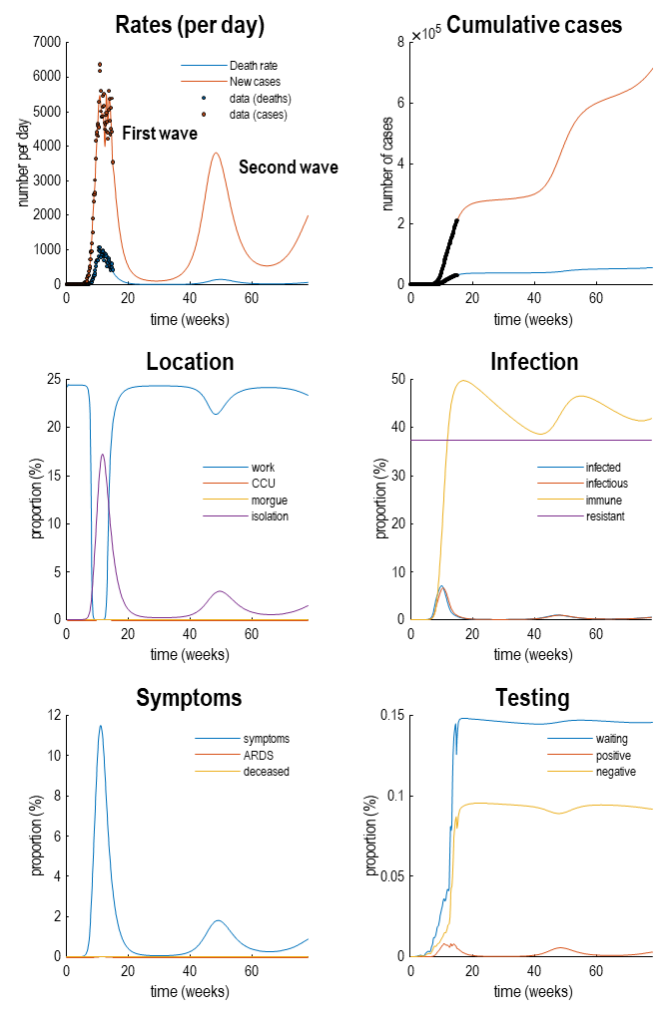
Latent causes. This figure shows the same data (with dots) and posterior expectations (solid lines) as in the previous figure over an 18-month period. However, here, it is supplemented with the underlying latent causes or expected states in the lower four panels. These constitute the hidden states that generate the outcomes in the upper two panels. The solid lines are colour-coded and correspond to the states of the four factors in Figure 1. For example, under the *location* factor, the probability of being found at work declined steeply from about 20% to 0 at the onset of the outbreak. At this time, the probability of isolating oneself rises to about 15% during the peak of the pandemic. After about five weeks, the implicit social distancing starts to relax and slowly tails off, with accompanying morbidity (in terms of symptoms) and mortality (in terms of death rate). As herd immunity (yellow line in the *infection* panel) declines the prevalence of infection accelerates to generate a second wave that peaks at about 48 weeks.

Figure 4 reproduces the results above (in the upper panels) and supplements these outcomes with the latent causes or hidden states that correspond to the factors in Figure 1. Here, we have shown the course of the pandemic over 18 months, as opposed to a six-month period. This illustrates the basic anatomy of the pandemic with an initial first wave, followed by a second wave some 36 weeks (nine months) later. The timing of this second wave depends upon the rate at which immunity is lost. In these simulations, a 16-month period of immunity was assumed.

Focusing on the initial outbreak (i.e., first wave), we can see the effects of social distancing as manifest in a very small probability of being found at work during the period of lockdown (blue line in the location panel). This coincides with a large number of people self-isolating (about 60% at its highest) during this period (purple line in the *location* panel). Under this model, we are currently experiencing the relaxation of social distancing, with a partial return to the pre-pandemic probability of being found at work. However, the world to which we return is differs from before the lockdown. This is because a substantial number of people have acquired immunity, in virtue of being infected (whether or not they show any subsequent symptoms). The acquisition of herd immunity is depicted by the yellow line in the *infection* panel. Although there are no data that inform these estimates, equivalent data from Germany is starting to appear. We will return to this later. Notice that a substantial proportion of the population (about 38%) have been estimated to be resistant. In other words, they have geographical or host factors that render them incapable of participating in the pandemic. For example, they may be isolated geographically ^6^ or may have genetic or developmental factors that preclude infection and viral shedding. In terms of *symptoms*, the most prevalent expression of the pandemic is in terms of symptoms that accompany an increase in self-isolation—and pre-empt a small number of people who go on to develop a severe syndrome (e.g., acute respiratory distress) from which they may not recover. The *testing* panel shows the progressive increase in people waiting to be tested (blue line) that subsumes people who are subsequently positive and negative. Initially, the number of negative tests is about twice the number of positive tests, but this ratio increases as the number of infected people in the population declines. The question is: what would happen to these trajectories under different monitoring or testing policies over the next few months?

**Figure 5.**
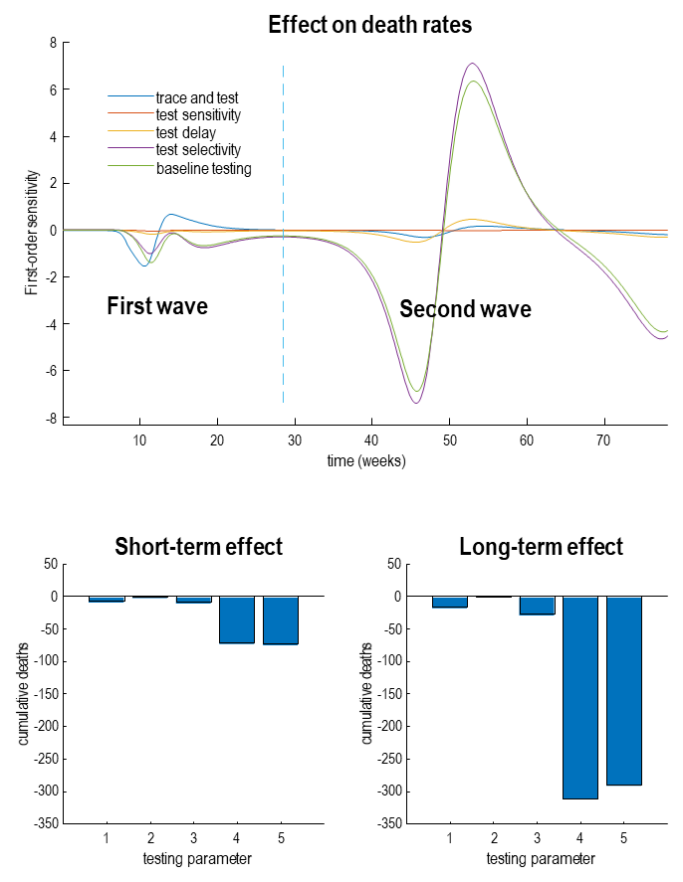
Sensitivity analysis. This figure illustrates the influence on death rates of various parameters that model diagnostic surveillance or testing. The upper panel shows the change in death rates with respect to the logarithm of the parameters controlling the efficacy of tracing and tracking (blue line) PCR testing in response to increasing levels of infection (red line), delay in reporting test results (yellow line), the selectivity for people who are infected (purple line) and baseline testing (green line). The lower panel shows the cumulative sensitivities or changes over time. The lower left panel sums these changes over a 20-week period prior to the onset of the outbreak, while the lower right panel accumulates the derivatives over a period of 18 months. The key thing to observe here is that the effect of changing testing or surveillance parameters is more marked during the second wave, relative to the first. Furthermore, note that the change in accumulated deaths, with respect to a unit change in log parameters is very small (in the hundreds as opposed to the thousands). This reflects the fact that the effect of these testing parameters is to shift the curve, not to attenuate its amplitude.

Figure 5 provides an analytic answer to this question in terms of the effects on daily death rates—as a function of time—as testing parameters are adjusted. Here, we enhanced the parameters that underwrite tracing and tracking (blue line), testing sensitivity (red line), testing delay (yellow line), testing selectivity for infected people (purple line) and, finally, the baseline probability of being testing (green line). The upper panel shows the effect on daily deaths when each of these parameters is increased by a scaling factor of one natural unit (i.e., 2.72). The key thing to observe is that the effect of changing these parameters itself changes over time. Here, we considered a period of 18 months; under the assumption that by the end of this period, there will be an effective vaccination program or other therapeutic advances.

There are two key things to note from this sensitivity analysis. First, the effect of any testing parameter on the first peak (before the vertical blue line), relative to the second peak. This second peak emerges because of a loss of immunity, modelled here with an immune period of 16 months (see Table 1). The second thing to note is that the effects are biphasic in nature. For example, increasing baseline testing initially decreases death rates with both the first and second waves, but increases death rates after the waves peak. At first glance, this may seem counterintuitive; however, there is a simple explanation. This rests on the fact that any surveillance measure has the effect of delaying the spread of the virus, such that the onset of successive waves of infection is suppressed and the peak is deferred or pushed into the future. In other words, increased surveillance affords the opportunity to reduce the spread of the virus, such that successive waves of infection are delayed and dispersed—along the lines of the ‘curve flattening’ notion. Indeed, this was the primary motivation for social isolation to avoid excess mortality due to a saturating clinical care capacity. However, in the absence of any limitations on critical care, surveillance, in and of itself, cannot attenuate the eventual spread of the virus throughout the population—it can only delay the spread. Metaphorically, this process can be imagined as rain falling from clouds. Eventually, the downfall will reach the sea. The only thing that one can do is to moderate the flow of water and mitigate against flood damage.

Quantitatively, this key point is reflected in the overall number of lives that will be saved by enhancing one aspect of surveillance or another. The lower panels in Figure 5 shows the cumulative number of lives saved under the five different parametric enhancements. As might be expected, increasing surveillance in various ways generally decreases the cumulative deaths; however, these effects are quantitatively very small (in the tens for an effect after the first wave (left panel) and in the hundreds after the second wave (right panel). This suggests that the utility of enhanced surveillance (e.g., tracing and tracking) can only be manifest if the second wave is pushed sufficiently far into the future that it is rendered innocuous through vaccination or other therapeutic interventions.

**Figure 6.**
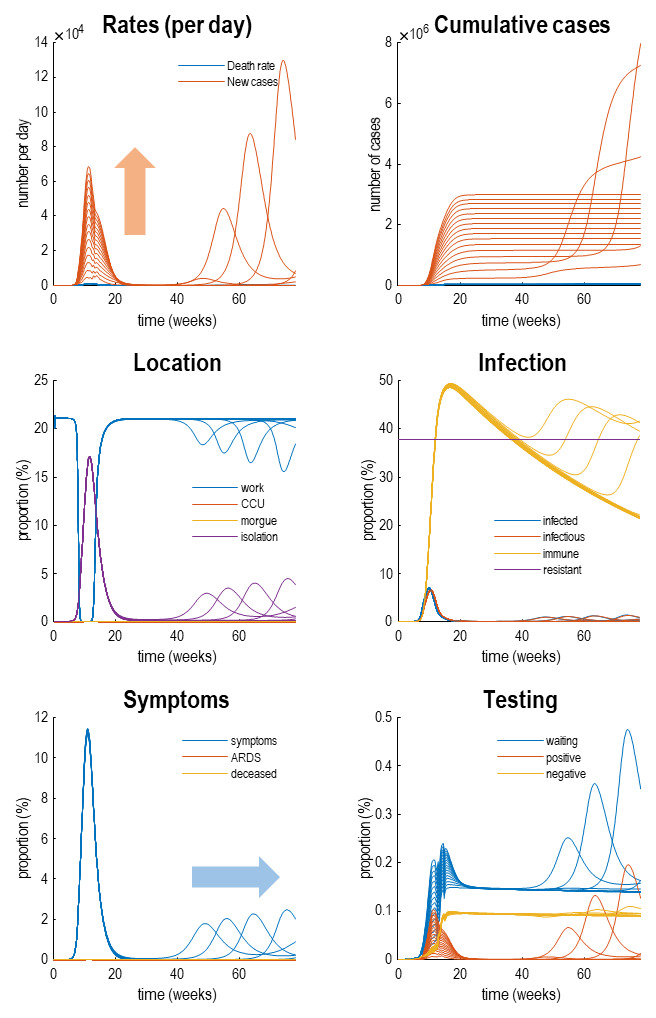
Testing and tracking. This figure reports the results of simulations under different protocols for testing and tracking. Specifically, we increased the probability of testing people who were infected but asymptomatic after the first wave. This increase in efficacy was from 0 to 1 in 32 steps. The results are shown using the format of Figure 4, for every fourth step. The key thing to take from this figure is that as one increases the efficacy of testing and tracking, the second wave is deferred or postponed beyond a time horizon (here, 18 months). At the same time, the total number of detected (positive) cases per day increases. These effects are summarised by the blue and orange arrows, respectively.

This is illustrated in Figure 6 which simulates the trajectories that one might expect when increasingly efficacy of testing and tracking (see the Methods for how efficacy is parameterised). This figure uses the same format as Figure 4 but reproduces trajectories under increasing levels of testing and tracking. In brief, one can see is that there is hardly any effect on the first wave in terms of social distancing ( *location*), prevalence of infection ( *infection*), or morbidity ( *symptoms*). However, the peak of the second wave is shifted progressively later in time, until it disappears beyond the 18-month time horizon simulated here. These simulations, as noted above, used a loss of immunity with a time constant of 16 months. This may be a somewhat pessimistic estimate of the rate at which we lose immunity; however, it clearly demonstrates the utility of testing and tracking under this (arguably worst-case) scenario. In summary, as the efficacy of testing and tracking increases, the second wave is progressively deferred, and the number of positive cases detected in the population rises. These effects are highlighted with the blue and orange arrows. So, what levels of testing and tracking would be necessary to preclude a second wave within a time horizon of 18 months?

**Figure 7.**
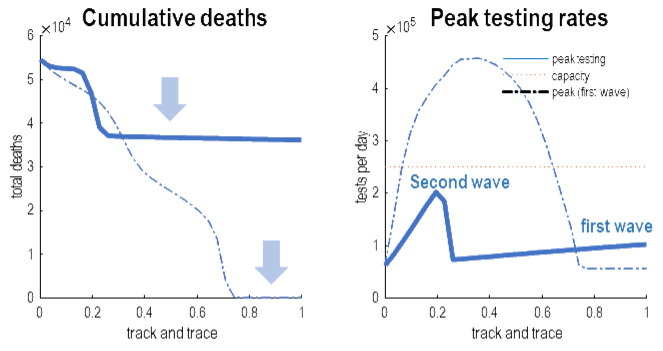
Suppressing waves. This figure summarises the results of the previous figure in terms of cumulative deaths after an 18-month period, as a function of the efficacy of testing and tracking. The left panel shows the total number of deaths as a function of the efficacy of a FTTI protocol that starts after the first wave (solid line) or before the first wave (broken line). The equivalent results are shown in the right panel in terms of the requisite peak testing rates over the course of the outbreak. The important thing to note is that that many lives, in principle, could be saved by eluding a second wave, provided the efficacy of FTTI exceeds about 24%. This is sufficient to defer or delay the second wave until it can be rendered innocuous (e.g. through the deployment of an efficacious vaccine). The suppression of the second wave is shown by the upper blue arrow. The lower blue arrow highlights the equivalent effect on the first wave had testing and tracking been implemented at the onset of the outbreak—and maintained at efficacy levels of over 70%. However, the requisite number of tests per day for intermediate level of efficacy may well have exceeded logistic capacity. This is illustrated by the red line in the right panel (here, 250,000 tests per day). In short, if one had a very small country or exceedingly well developed FTTI resources, it would have been possible to eliminate the first wave; however, for a country like the United Kingdom, this would probably not have been a practical option.

Figure 7 answers this question by plotting the cumulative deaths and peak testing rates as a function of the efficacy of tracking and tracing. These posterior predictions were based on increasing the efficacy of testing and tracking from 0 to 1 in 32 steps—depicted every four steps in the previous figure. As the efficacy of tracking and tracing increases there is a marked reduction in cumulative deaths in the order of 10,000 people. This reflects the delay in the second wave (solid line). Quantitatively, it would be sufficient to have an efficacy of about 24% to defer the second wave until 18 months. According to these estimates, this would entail peak testing rates of less than 10,000 tests per day, well within the reach of current testing capacity.

The dotted lines show the corresponding predictions for a testing and tracking strategy that was instantiated prior to the first wave. These results are interesting in the sense they speak to what would have happened had the UK government pursued a testing and tracking strategy at the onset of the pandemic. In principle, there was a potential to defer the first wave and thereby elude any deaths due to COVID-19. This is shown by the second arrow in the left panel of Figure 7. However, things are not quite that simple. In order to eliminate the first wave, it would have been necessary to have an efficacy of testing and tracking of about 80% or more. In other words, nearly everybody who was infected but asymptomatic would have to have been identified. A more realistic efficacy of 50% would have reduced deaths in the initial phases of the outbreak; however, this would have required peak testing rates beyond the capacity of a country like the United Kingdom. This is illustrated by the dashed line in right panel that surpasses an arbitrary threshold of 250,000 tests a day. In short, although a suppression strategy based on testing and tracking is a theoretical possibility, it cannot be realised after the number of infected people exceeds testing capacity. It is interesting to speculate what this means for countries like South Korea and Singapore who have managed to elude a substantive first wave. In virtue of the fact that they have not acquired a meaningful herd immunity, they may have to maintain a high level of efficacy of FTTI, in conjunction with strict border controls and accompanying quarantine. From this perspective of the United Kingdom, the question is: do the same mechanics of surveillance apply to the second wave?

**Figure 8.**
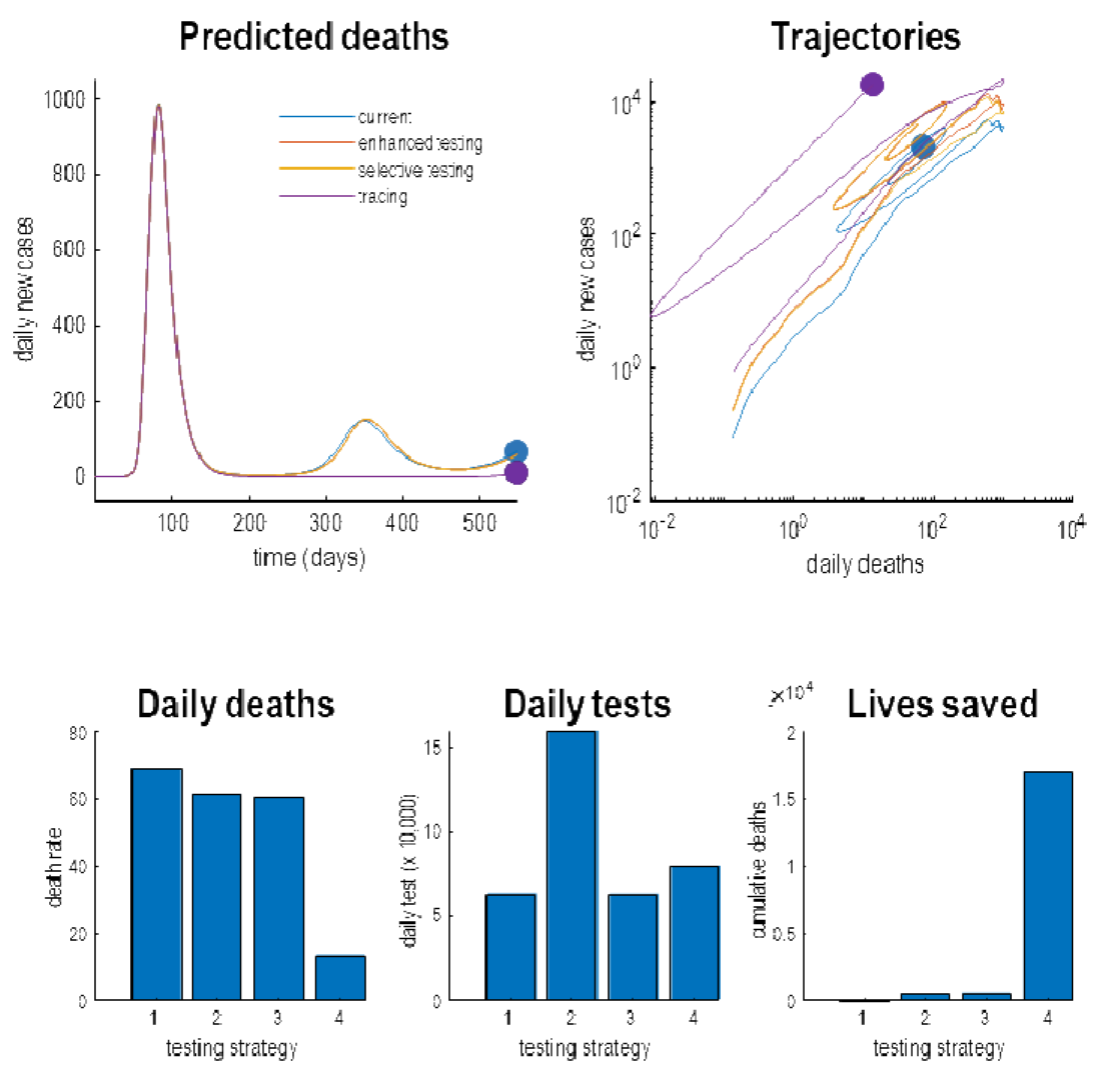
Different testing strategies. This figure reports simulations of what might happen under different testing or surveillance regimes. The upper panels show the simulated daily deaths predicted under four strategies. These include the current strategy based upon the posterior expectations of testing parameters. The red lines show the response to an enhanced baseline testing. The yellow lines simulate the outcomes under an increase in selective testing, while the green lines illustrate the impact of testing and tracking within efficacy of 25%. For the enhanced testing, the posterior expectations of the log parameters were increased by a value of one (i.e., the scale parameters were multiplied by 2.72). The FTTI parameter was increased to a value of one quarter. The upper left panel plots the predicted deaths as a function of time for an 18-month period. The upper right panel shows the same data but plotted as a trajectory in a phase space, spanned by daily deaths and reports of new cases. This illustrates the fact that, mathematically, the trajectories into the future correspond to flows towards an attracting orbit or set. In this instance, the systems have point attractors. However, here, we have assumed that a vaccine is available at 18 months, at which point the trajectories terminate in the filled circles. The lower three panels characterise this endpoint in terms of daily deaths, daily tests and total number of lives saved since the onset of the outbreak. It can be seen that, as might be anticipated, the successive enhancements of testing reduce daily deaths. The most expensive strategy, in terms of daily tests, is the enhanced testing strategy requiring 160,000 tests again. The remaining strategies require a more modest 70,000 tests per day. The most efficient and life-saving strategy is the implementation of testing and tracking that could, under this model, said more than 15,000 lives. The blue dots in the upper panels denote the endpoints that, here, stand in for the endemic equilibria under the four strategies. Note that the testing and tracking strategy is the only its approach that materially decreases daily deaths, both over time and at the endpoint.

The answer to this question is no. This is because the context in which the second wave manifests is very different from the first wave. This follows because of the acquisition of herd immunity, which means that the spread of the virus—in the run-up to the second wave—is substantially attenuated. In turn, this means that the requisite efficacy of FTTI is substantially smaller. This point is illustrated in Figure 8 by evaluating the predicted outcomes at the (18-month) time horizon under four scenarios. The first scenario was a scenario based upon the posterior estimates of the testing parameters. The second scenario entailed an enhanced baseline testing. The third scenario was an enhancement of selective testing; namely, increasing the relative probability of testing infected people. Finally, we consider a FTTI strategy, in which the efficacy was increased from near zero to 25%. The upper panels show the posterior predictions as a function of time (left panel) and as a phase-space summary of the same trajectories (right). This way of detecting trajectories plots one outcome against another: i.e., plotting the daily rates of new cases against daily deaths.

The resulting trajectories illustrate effects of various interventions. In brief, as we increase the rate or selectivity of testing, we shift the trajectories upwards. In other words, we increase the number of detected cases but with little effect on daily deaths. In contrast, the FTTI strategy suppresses the second wave and reduces daily deaths. The lower panel quantifies the endemic endpoint at the time horizon of 18 months. This is not an endemic equilibrium but stands in for the state of affairs at the point of a presumed vaccination or therapeutic intervention. One can see that the various testing strategies progressively reduce the daily death rates at this endpoint. For example, with an FTTI efficacy of 25%, the daily deaths due to COVID-19 are about 10 per day. This is roughly the number of people who are killed in road traffic accidents ^7^. The number of tests at this time, are reasonably manageable (about 50,000 per day for the FTTI strategy). Crucially, the number of lives saved is reduced considerably under, and only under, FTTI. In this example, the elimination of the second wave would save about 16,000 lives. Notice that simply elevating the sensitivity or selectivity of testing has little effect on the mortality rates. Only the FTTI strategy enables the early identification of infected individuals, their subsequent isolation and ensuing deferment of a putative second wave.

An important (if obvious) observation, implicit in this treatment, is that testing can be deployed in different ways with distinct agendas. Crucially, the only kind of testing that matters for saving lives is identifying those individuals who are infected before they can spread the virus. This is the *raison d'être* for FTTI, as opposed to simply increasing test rates. Increasing the baseline, sensitivity or selectivity of testing provides more precise data for epidemiological modelling and subsequent policy decisions; however, in and of itself, it will not have any material effect on the progression of the pandemic. Similarly, testing people who are symptomatic is too late from the point of view of isolating individuals who may become contagious—even if it allows people to return to work early. Clearly, all three agendas are important; however, it may be useful to consider (and model) testing in terms of its distinct aims; namely, to defer a second wave, to enhance epidemiological surveillance and to ease pressure on the economy and clinical care.

### A comparative analysis

**Figure 9.**
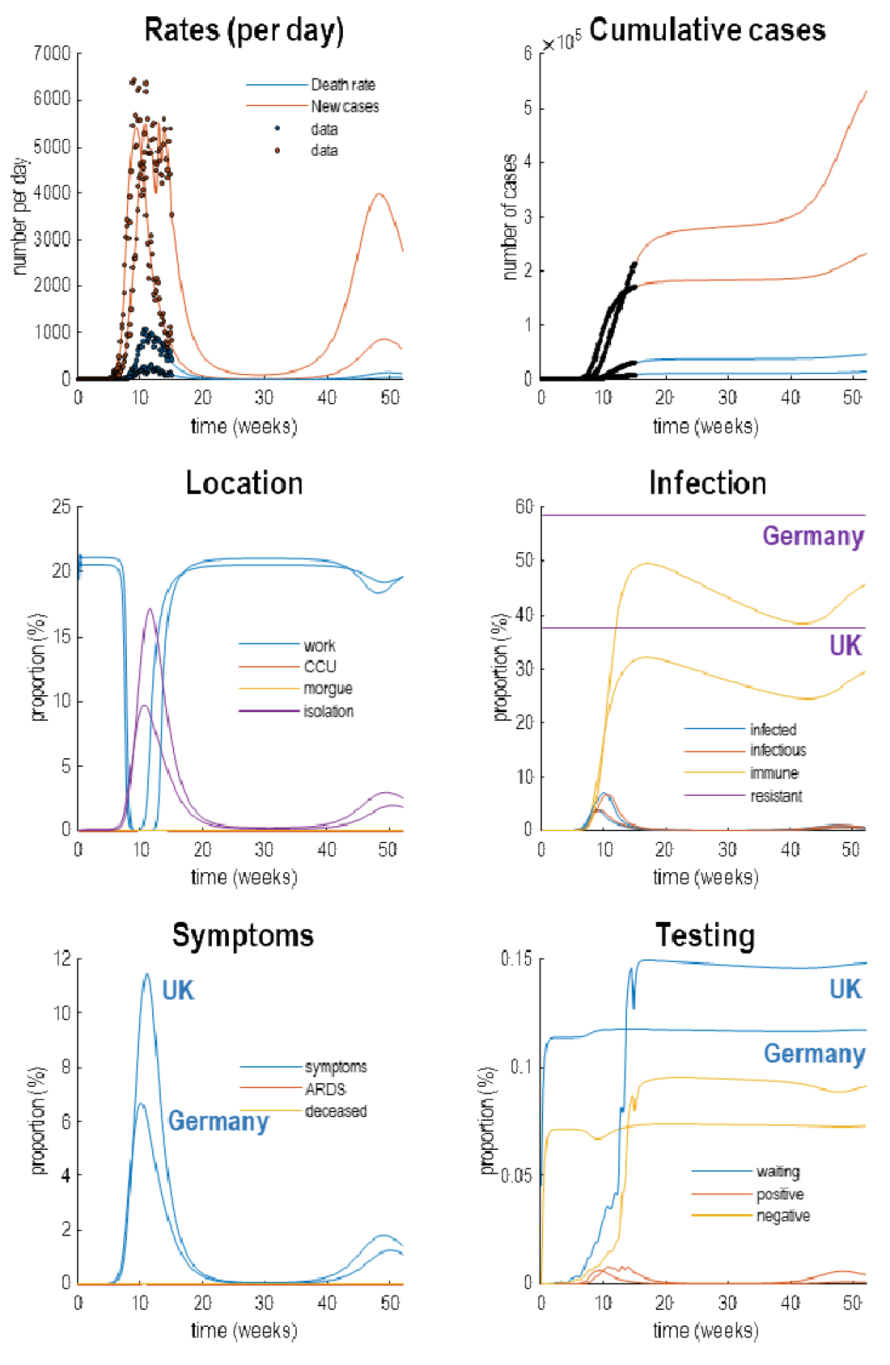
Germany and the UK compared. This figure shows the latent causes (lower panels) of observed and predicted outcomes (upper panels) for Germany and the UK. The coloured pairs of lines in each panel use the same format as Figure 4 and refer to the two countries in question. The generative model provides a good account of the empirical data for both countries (black dots in the upper panels), with formally similar fluctuations in latent epidemiological states. However, there are some key quantitative differences. For example, the degree of self-isolation and social distancing is attenuated by roughly one half in Germany. This pertains both to the percentage of people self-isolating and the duration of social distancing at a societal level. This is also reflected in the lower prevalence of symptomatic individuals at the first (and second) peaks of infection. These differences are, in large part, due to the number of people who are susceptible to infection, as reflected in the proportion of people who are resistant (about 38% for the UK and 58% for Germany). The parameters that underwrite these trajectories are shown in the next figure.

The conclusions from the above modelling are clear. There is an imperative to instantiate (or possibly re-instantiate) FTTI at modest levels of efficacy in the next few months—to preclude a second wave by delaying it. Furthermore, trying to maintain an early FTTI strategy at effective levels would have been logistically difficult. This begs the question: how has Germany managed to suppress its mortality rates, if it contended with the same kind of outbreak confronting the United Kingdom? To answer this question—at the fairly crude level—we repeated the dynamic causal modelling using daily new cases and deaths from Germany. The trajectories of these outcomes and their latent causes are shown in the upper and lower panels of Figure 9, respectively. Because there was no available data on the total number of tests, we assumed a constant baseline testing.

The ensuing differences in the outcomes and latent states speak to what we already know. For example, despite having about the same number of people testing positive during the first peak, the mortality rates in Germany are about a quarter of those witnessed in the UK. The inferred surveillance and testing suggest that Germany started with a baseline testing rate, such that at any one time 0.1% of the population was waiting for their test results. The UK, conversely, accrued its testing capacity during the first wave and, according to these estimates, now exceeds the German testing rates. Despite increased testing in Germany, the number of people self-isolating was about half that in the UK (as estimated under this model), with less than 10% of the German population quarantining themselves at the peak of the pandemic. Furthermore, Germany's social distancing was less stringent and shorter, as reflected by the blue lines in the location panel of Figure 9. The infection panel is telling; in the sense that about 38% of the UK population are estimated to be resistant. However, this rises to about 58% of the German population. This is a marked difference suggesting either geographical or host factors may play an important role in the differential fatality rates. Indeed, when one examines the underlying posterior parameter estimates for the trajectories depicted in Figure 9, it becomes clearer how Germany and the UK differ.

**Figure 10.**
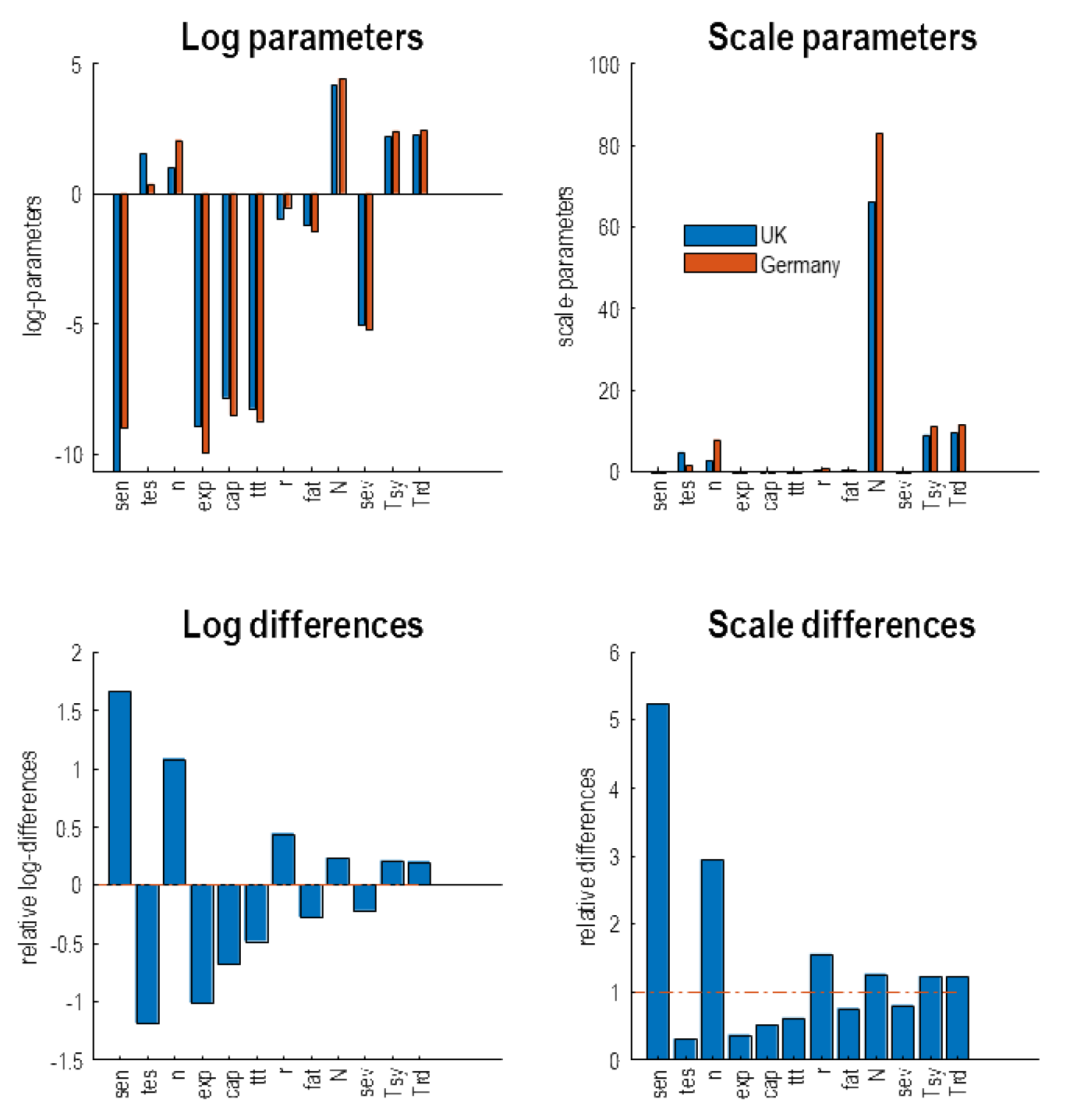
A parametric comparison. This figure shows the posterior expectations of the model parameters form Germany and the UK. The upper panels show the estimates for each country separately, while the lower panels show the differences. The left panels show the parameters in terms of their logarithmic form, while the right panels show the exponentiated (scale) parameters. In the upper panels, the UK parameters are in blue and the German parameters are in orange. The labels on the X axis correspond to the parameter subscripts in Table 1. The key thing to take from this figure is that the most marked (quantitative) differences between the two countries lies in the parameters pertaining to testing; namely, the sensitivity to the prevalence of infection, the selectivity of testing in terms of whether people are infected or not and the sustained testing after the first peak. Having said this, testing and tracking is actually less in Germany—as estimated by the model—than in the UK, while Germany looks (i.e., appears) as if it has a more sensitive testing or surveillance program that is less selective for people who are infected. These testing parameter differences explain why the number of positive cases reported is about the same for United Kingdom and Germany, while the latent number of people who are infected and subsequently die is much less (by a factor of roughly 4). The actual cause, according to this model, of this differential mortality lies in the clinical and management parameters. These include an increased number of resistant members of the population and a reduced fatality rate, when severely ill. Furthermore, there is a decrease probability of developing severe symptoms when infected.

Figure 10 shows the parameters with the greatest difference between Germany and the UK, in terms of the country specific estimates (upper panels) and the differences (lower panels). The parameters are shown in terms of log parameters (left panels) and the corresponding scale parameters (right panels). The scale parameters are nonnegative rate constants and probabilities, while the log parameters are simply the log transformed scale parameters. For clarity, only the 12 parameters with the greatest posterior difference are shown. They have been ranked such that the parameters on the left show the greatest difference (the parameters are labelled by the subscripts in Table 1). The key thing to take from this comparison is that there are marked differences between Germany and the UK, both in the testing parameters and the parameters pertaining to susceptibility and clinical surveillance. Indeed, the most marked difference is a fivefold increase in the sensitivity of German testing to the prevalence of infection. This testing is nearly 5 times less selective for infected people than in the UK. This is consistent with what we know from the German approach relative to the U.K.'s approach. The third largest difference is the number of people infected ( *n*) at the beginning of the timeseries. The inference here is that Germany started with about three times as many infected people as the United Kingdom. By virtue of the fact that Germany tested more sensitively but non-selectively from the onset of the outbreak, the sustained testing component ( *exp*) is much smaller. Note also that the FTTI parameter ( *ttt*) is also smaller. In other words, there is no evidence that testing and tracking in Germany contributed to their surveillance program. The key parameters to note here are the substantial (about 50%) increase in the proportion of the German population that were resistant to infection and a (about 20%) decrease in the probability of fatality in critical care. This is despite Germany having a larger population (83 million as opposed to 66 million in the United Kingdom). Finally, the probability of developing severe symptoms when infected is slightly lower than in the UK.

The evidence here contradicts the hypothesis that Germany's relatively low fatalities are caused by a more vigorous testing programme. At one level, this hypothesis is naive because testing cannot cause morbidity. It can only detect the consequences of morbidity. As noted above, the only way that testing can affect fatalities is by delaying the spread of the virus—and this is most effective when infected but asymptomatic individuals are identified through testing and tracking. A more plausible interpretation of these parameter estimates, and ensuing predictions is as follows:

The proportion of people testing positive who subsequently died in Germany is lower not because people who are infected are less likely to die but simply because Germany has tested more people. The reduced mortality rates may reflect the differential prevalence of infection in cohorts of the population that are more resistant. The relative reduction in the probability of developing severe symptoms and subsequent fatality may well reflect the clinical surveillance and management of symptomatic people. For example, anecdotal reports from respiratory physicians in Germany suggest a more prospective clinical management, with lower thresholds for admission to critical care. In contrast, much of the disease burden in the UK appears to have been managed in an elderly and vulnerable population in care homes. This cohort are unlikely to survive the rigours of intubation in an intensive care unit and their clinical management is necessarily more palliative. In short, Germany may have had to deal with a different kind of problem than that confronting the UK. In short, although German testing and clinical surveillance was more in evidence, only the clinical surveillance mattered in terms of mortality.

Clearly, this is purely speculation; however, in principle, it should be possible to evaluate the evidence for these speculative hypotheses when more detailed data becomes available. This speaks to one application of dynamic causal modelling to compare different models in terms of their evidence ( Penny *et al.*, 2004); for example, comparing a model of outcomes in Germany and the UK with, and without, country-specific differences in the parameters.

## Conclusion

The key conclusions from this kind of modelling are twofold. First, the hypothesis that a flareup or rebound of infections will ensue if we relax social distancing prematurely are not supported by the evidence at hand. A popular conception of this immediate (second wave) is akin to lowering the ‘flood gates’ too soon and being overwhelmed with a deluge of infections. However, this picture may be a false impression. There can be no flood of infections because a sufficient proportion of the population have already been exposed to the virus. These people preclude a rapid spread of the virus through the population by acting as a retardant or buffer that suppresses the effective reproduction ratio. In other words, the first wave cannot flareup because it has largely exhausted the necessary substrate of susceptible individuals it needs to disseminate itself. This speaks to the second key conclusion.

Over the forthcoming months any ‘flood’ will be a ‘trickle’. In this limited window of opportunity, FTTI protocols become viable, in the sense that detecting asymptomatic and infected individuals with a reasonable (e.g., 25%) efficacy would be sufficient to delay the re-emergence of the virus—a re-emergence that rests on, and only on, a slow loss of immunity. In short, FTTI will work after the first wave, even if it was logistically viable before the first wave. If, collectively, we lose immunity over a period of many months or years, then a second wave can be eluded, via FTTI, saving thousands of lives. This is under the proviso that a second wave can be pushed sufficiently far into the future, where it is rendered innocuous by vaccination or other interventions.

**Figure 11.**
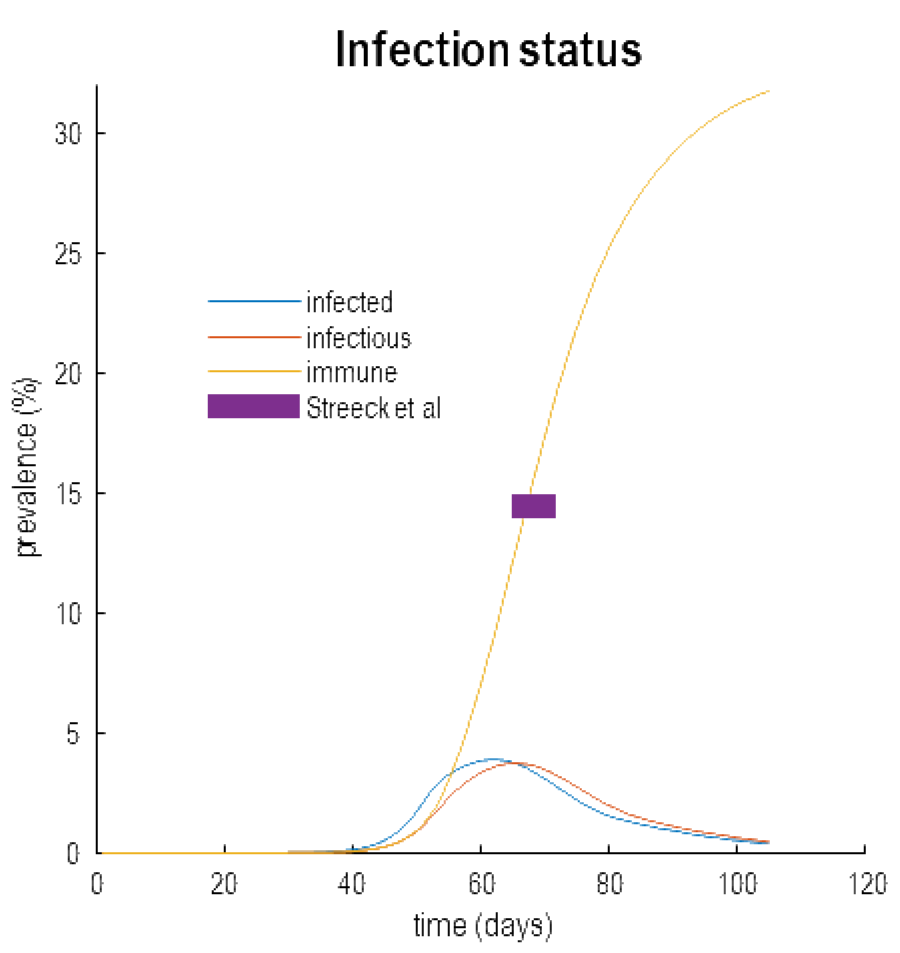
Herd immunity. This figure reproduces the results of Figure 9 but with a focus on infectious status. Here, the estimated prevalence of infected people, contagious ( *infectious*) people and immune people are shown as a function of time since the onset of the outbreak (25th of January 2020). The purple bar indicates the level of estimated seroprevalence in a region in Germany as reported in streak *et al.* This matches the predictions of the model; namely, a 15% herd immunity at the peak of the infection. According to this analysis, this level of herd immunity is contextualised by the proportion (about 42%) of the population that are susceptible to infection and subsequent morbidity.

Clearly, this narrative depends on the acquisition of (herd) immunity following the first wave and its subsequent loss due to population fluxes, geo-social and serological factors. The big question at the moment is whether the first wave has induced a sufficient level of herd immunity to open the window of opportunity for testing and tracking. Although there is no current data for the UK, early studies in Germany speak directly to this issue. Figure 11 reproduces the hidden states in Figure 9, with a focus on infection status. The prevalence of immunity is shown as a yellow line. At the peak of the first wave (shortly after 60 days) the inferred level of immunity is 15%. This is the level of immunity estimated empirically in provisional reports of a serological study of people living in a region near Bonn ( Streeck *et al.*, 2020) ^8^. Results of this sort are encouraging and endorse the inferences afforded by dynamic causal modelling. Perhaps more importantly, over the next few weeks more serological studies will become available and we will know with much greater certainty whether the above narrative is licensed by empirical data. If not, these data can be assimilated into the model to update our (Bayesian) beliefs about what has happened, what will happen and what could happen.

## Methods

### Modelling self-isolation

Equipped with an extra location ( *isolation*) state enables one to distinguish between simply staying at home or being out and about (i.e., at *work*). These states are rough approximations to the different kinds of environment we find ourselves in and are used to differentiate the number of contacts that could potentially transmit the virus from one person to another. When considering the parameterisation of population dynamics, in terms of being in a particular state, one has to parameterise the time spent in that state, in relation to the probability of leaving or entering it. In this instance, the probability of entering self-isolation is unity when you develop symptoms or submit to PCR testing. You then remain in that state for seven days, unless you receive notification that you have tested negative. While in this state, you can neither infect nor be infected by anybody else. Mathematically, this can be parameterised as follows, where *τ _iso_* is seven days (conditional on not being in critical care or the morgue)

$$P\left(isolation_{t+1}|isolation_{t}\right)=\{\begin{array}{cc} 1 & \text{if}\;\text{sympotomatic,}\;\text{positive}\;\text{or}\;\text{waiting}\\ 0 & \text{if}\;\text{negative}\;\text{and}\;\text{asymptomatic}\\ \text{exp}\left(-1/\tau_{iso}\right) & \text{otherwise} \end{array}\;(1.1)$$

Clearly, the transition from one *location* state to another now depends upon the *testing* factor. If you are *waiting* for a test, you move into *isolation* and if you are *negative* you leave. It is this conditional dependency between the factors that mediates the efficacy of FTTI.

### Modelling FTTI

The parameterisation of FTTI and other *testing* state transitions is a bit more delicate. This is because there are several reasons you might be tested that depend on several factors. In this model, we parameterise testing by first establishing a time-dependent probability of being tested on any given day. If we now condition the probability of being tested on whether or not you are infected, we need to parameterise the relative probability of being tested when an infected, in relation to not being infected. This means that the total probability of being tested is a weighted average of two probabilities, parameterised as follows (Please see Table 1 for a description of the parameters):

$$\begin{array}{l} P\left(tested\right)=P\left(tested|infected\right)P\left(infected\right)+P\left(tested|not\;\;infected\right)P\left(not\;\;infected\right)\\ \;\;\;\;\;\;\;\;\;\;\;\;\;\;\;\;\;\;\;\;\;\;\;\;\;\;\Rightarrow \\ \;\;\;\;\;\;\;\;\;\;\;\;\;\;\;\;P_{tes}=P\left(tested|infected\right)=\theta_{tes}P_{sen}\\ \;\;\;\;\;\;\;\;\;\;\;\;\;\;\;P_{sen}=P\left(tested|not\;\;infected\right)=\frac{P\left(tested\right)}{\theta_{tes}P\left(infected\right)+1-P\left(infected\right)} \end{array}\;(1.2)$$

where the probability of being tested has three components:

$$P(tested)=\theta_{bas}+\theta_{sen}P\left(infected\right)+\theta_{\text{exp}}P\left(immune\right)\;(1.3)$$

The first is a *baseline* probability. For the UK analyses, this parameter was proportional to the total number of tests up until the present time, and the maximum number of tests in the future. The second parameterises a *sensitivity* to the prevalence of infection in the community and increases with infection rates. The final term is a *sustained* response following onset of the first wave. Here, we use the level of immunity as a proxy for a gently declining function of the cumulative number of affected people. Each of these terms has a parameter enabling one to fit a time-dependent probability of being tested. Finally, we have to consider targeted testing of individuals who have been identified as having been in contact with an infected individual. This affords an enhanced probability of testing if, and only if, you are infected and asymptomatic. By adding this probability to the probability of being tested when infected, we supplement general screening with a FTTI parameter as follows:

$$P\left(tested|infected,asymptomatic\right)=P_{tes}+\theta_{ttt}\left(1-P_{tes}\right)\;(1.4)$$

The ensuing parameter is simply the efficacy or extra probability that I will be tested if I am infected and asymptomatic. If it were possible to trace and test everybody who has been exposed and contracted the virus prior to developing symptoms, this efficacy will be one. In the absence of any targeted testing efficacy will be zero. A priori, the efficacy was set to very low levels of one in 10,000 people, per day.

### Effective reproduction rate

The effectively production rate is a fundamental epidemiological constant that provides a useful statistic that reflects the exponential growth of the prevalence of infection. There are several ways in which it can be formulated and estimated. For our purposes, we can generate an instantaneous reproduction rate directly from the time varying prevalence of infection as follows:

$$\begin{array}{l} R_{t}=\text{exp}\left(K_{t}\cdot \tau_{con}\right)\\ K_{t}=\text{In}\frac{P\left(infected_{t+1}\right)}{P\left(infected_{t}\right)}=\frac{\text{In}\left(2\right)}{T_{d}} \end{array}\;(1.5)$$

These expressions show that the reproduction rate reflects the growth of the (logarithm of) proportion of people infected—and the period of being infectious. This number is formally related to the doubling time *T _d_*. Note that the reproduction rate is not an estimate in this scheme: it is an outcome that is generated by the latent causes or hidden states inferred by inverting (i.e., fitting) the model to empirical timeseries.

### Software note

The annotated (MATLAB/Octave) code is available as part of the free and open source academic software SPM ( https://www.fil.ion.ucl.ac.uk/spm/), released under the terms of the GNU General Public License version 2 or later. The routines are called by a demonstration script that can be invoked by typing DEM_COVID_T at the MATLAB prompt. For this technical report, we used MATLAB R2019b and SPM12 r7850 (archived at https://doi.org/10.6084/m9.figshare.12174006.v4 ( Friston *et al.*, 2020c)).

We recommend anyone interested in applying this model should use the latest version of the software available. Details about future developments of the software will be available from https://www.fil.ion.ucl.ac.uk/spm/covid-19/.

### Software availability

Software is available from: https://www.fil.ion.ucl.ac.uk/spm/covid-19/

Archived source code at time of publication: https://doi.org/10.6084/m9.figshare.12174006.v4 ( Friston *et al.*, 2020c)

License: GLP 2.0+

## Data availability

### Source data

The data used in this technical report are available for academic research purposes from the COVID-19 Data Repository by the Center for Systems Science and Engineering (CSSE) at Johns Hopkins University, hosted on GitHub at https://github.com/CSSEGISandData/COVID-19 and from the Coronavirus (COVID-19) UK Historical Data repository by Tom White hosted on GitHub at https://github.com/tomwhite/covid-19-uk-data and also from figshare.

### Underlying data

Figshare: Dynamic Causal Modelling of COVID-19. https://doi.org/10.6084/m9.figshare.12174006.v4 ( Friston *et al.*, 2020c)

This project contains the following underlying data:

- covid-19-tests-uk.csv (UK COVID-19 historical data)

Data are available alongside the source code under a GLP 2.0+ license.

## Footnotes

^1^ Shedding of COVID-19 viral RNA from sputum can outlast the end of symptoms. Seroconversion occurs after 6–12 days but is not necessarily followed by a rapid decline of viral load.

^2^ Available from https://github.com/CSSEGISandData/COVID-19.

^3^ Available from https://github.com/tomwhite/covid-19-uk-data.

^4^ Public Health England estimates that on average 17,000 people have died from the flu in England annually between 2014/15 and 2018/19. However, yearly deaths vary widely, from a high of 28,330 in 2014/15 to a low of 1,692 in 2018/19 (dashed red lines in Figure 2). Please see Public Health England annual flu reports here: https://www.gov.uk/government/statistics/annual-flu-reports

^5^ Available from https://mrc-ide.github.io/covid19estimates/#/download.

^6^ See https://www.esade.edu/itemsweb/wi/research/ecpol/EsadeEcPol_Insigth6_Exit_Strategy.pf for a discussion of isolation in terms of social networks and green zones.

^7^ https://www.gov.uk/government/collections/road-accidents-and-safety-statistics.

^8^ Preliminary results from a town of about 12,500 in Heinsberg—a region in Germany that had been hit hard by COVID-19—suggest a seropositive prevalence of 14% of 1007 people assessed between March 30 and April 6 2020.
