# Supplementary material for: Testing and tracking in the UK: A dynamic causal modelling study
Source: Wellcome Open Res. 2021 Feb 15;5:144. Originally published 2020 Jun 17. [Version 2] doi: 10.12688/wellcomeopenres.16004.2 (PMC13231967; doi:10.12688/wellcomeopenres.16004.2)
Supplement: Peer review history

# Reviewer report: Reviewer response for version 2

**DOI:** 10.21956/wellcomeopenres.18339.r156222

**Published:** 2026-06-02

**Authors:** Fahad Mostafa

**Affiliations:** 1 Arizona State University, Tempe, USA

Manuscript presents an interesting and timely application of dynamic causal modelling (DCM) to evaluate the impact of testing, tracing, and tracking strategies on COVID-19 transmission in the UK.

 Paper is methodologically ambitious and provides a comprehensive framework integrating epidemiological dynamics, behavioral responses, and testing policies within a Bayesian inference framework. The comparison between the UK and Germany further strengthens the practical relevance of the work.

 The manuscript is generally well written and the simulations are informative; however, several assumptions of the model; particularly regarding immunity duration, resistance proportions, and homogeneous population mixing—require stronger justification and sensitivity analysis.

 In addition, some conclusions regarding the inevitability of second waves and the limited effect of testing on mortality may appear overly strong given uncertainties in real-world data and evolving viral dynamics. 

 However for consideration, the paper needs to address

1. How sensitive are the model predictions to the assumed duration of immunity (16 months), and were alternative immunity scenarios systematically evaluated?
2. The model assumes a substantial proportion of the population is “resistant.” What biological or epidemiological evidence supports this assumption, and how identifiable is this parameter from the available data?
3. Could the authors provide additional validation of the DCM forecasts against later observed epidemic waves or independent datasets?
4. The manuscript concludes that testing alone cannot substantially reduce mortality. How would this conclusion change under heterogeneous age structures, healthcare access, or vaccination rollout scenarios?
5. The comparison between the UK and Germany attributes mortality differences partly to resistance and clinical management. Could unmeasured confounders or differences in reporting practices also explain these findings?
6. The model does not explicitly include repeated importation/reseeding events or spatial heterogeneity. How might incorporating these mechanisms alter the predicted second-wave dynamics?
7. Can the authors clarify the practical interpretation of the estimated FTTI efficacy parameter and how it relates to measurable public health metrics in real-world tracing systems?

Is the rationale for developing the new method (or application) clearly explained?

Yes

Is the description of the method technically sound?

Yes

Are the conclusions about the method and its performance adequately supported by the findings presented in the article?

Yes

If any results are presented, are all the source data underlying the results available to ensure full reproducibility?

Yes

Are sufficient details provided to allow replication of the method development and its use by others?

Yes

Reviewer Expertise:

Biostatistics, Epidemiology, Biomedical Data Science, Public health

I confirm that I have read this submission and believe that I have an appropriate level of expertise to confirm that it is of an acceptable scientific standard.

# Reviewer report: Reviewer response for version 2

**DOI:** 10.21956/wellcomeopenres.18339.r42617

**Published:** 2021-04-12

**Authors:** Lin Wang

**Affiliations:** 1 Department of Genetics, University of Cambridge, Cambridge, UK

The authors have addressed my comments. My pleasure to suggest the acceptance.

Is the rationale for developing the new method (or application) clearly explained?

Partly

Is the description of the method technically sound?

Partly

Are the conclusions about the method and its performance adequately supported by the findings presented in the article?

Partly

If any results are presented, are all the source data underlying the results available to ensure full reproducibility?

Yes

Are sufficient details provided to allow replication of the method development and its use by others?

Partly

Reviewer Expertise:

Infectious diseases

I confirm that I have read this submission and believe that I have an appropriate level of expertise to confirm that it is of an acceptable scientific standard.

# Reviewer report: Reviewer response for version 1

**DOI:** 10.21956/wellcomeopenres.17553.r40101

**Published:** 2020-10-15

**Authors:** Lin Wang

**Affiliations:** 1 Department of Genetics, University of Cambridge, Cambridge, UK

In this manuscript, Friston and colleagues extended their established compartmental models to estimate the impact of testing and tracking policies in the UK. I'm a bit confused about their findings and conclusions. Please see below my comments:

1. The authors claimed that "under plausible (graded) relaxations of social distancing, a rebound of infections is highly unlikely. The emergence of a second wave depends almost exclusively on the rate at which we lose immunity". However, it is clear that the UK is having the second wave for the period explored in this study. So the model may contain some unrealistic assumptions or parameters.
2. In Table 1, the source of parameters is unknown. Which parameters were obtained from other studies? Which parameters were estimated in this study?
3. How the model was fitted to data? Are you using MCMC? MLE? EM?
4. The model does not include the influence of repeated seeding. For example, the UK has detected lots of introductions of SARS-CoV-2 cases, which is much higher than the assumed number of initial cases in this study. ^1^
5. The assumptions on contact patterns are oversimplified. Please account for age-stratified contact patterns. ^2^
6. The authors assumed that self-isolated cases could not infect others. This is somehow problematic. For example, if a case was isolated at home, he/she can infect household members.

Is the rationale for developing the new method (or application) clearly explained?

Partly

Is the description of the method technically sound?

Partly

Are the conclusions about the method and its performance adequately supported by the findings presented in the article?

Partly

If any results are presented, are all the source data underlying the results available to ensure full reproducibility?

Yes

Are sufficient details provided to allow replication of the method development and its use by others?

Partly

Reviewer Expertise:

Infectious diseases

I confirm that I have read this submission and believe that I have an appropriate level of expertise to state that I do not consider it to be of an acceptable scientific standard, for reasons outlined above.

## References

[1] : Using a real-world network to model localized COVID-19 control strategies. *Nat Med* .26(10) : 10.1038/s41591-020-1036-8 1616-1622 10.1038/s41591-020-1036-8 32770169

[2] : Preliminary analysis of SARS-CoV-2 importation and establishment of UK transmission lineages. Reference source

# Response

**Published:** 2021-02-04

**Authors:** Adeel Razi

**Affiliations:** Monash University, Australia

Dear Dr. Wang,

 We would like to thank you for the time and effort you have spent reviewing our manuscript. Below are the replies to the comments. We hope these revisions are what you had in mind.

- *The authors claimed that "under plausible (graded) relaxations of social distancing, a rebound of infections is highly unlikely. The emergence of a second wave depends almost exclusively on the rate at which we lose immunity". However, it is clear that the UK is having the second wave for the period explored in this study. So, the model may contain some unrealistic assumptions or parameters.*

Dr Wang raises an excellent point; namely that a rebound may occur in the absence of any material loss in immunity. We have used the dynamic causal model in this report to address the distinction between secondary or rebound waves of infection due to the spread of the virus from the initial population to other populations or communities—and how this is distinguished mechanistically from a second wave due to loss of effective immunity in the first population. A full treatment can be found in the following technical report and op-ed

- *In Table 1, the source of parameters is unknown. Which parameters were obtained from other studies? Which parameters were estimated in this study?*

In Table 1, we provide the list of the model parameters (second column) and their prior densities (their mean and variance in the 3 ^rd^ and 4 ^th^ column respectively). These are the model parameters which we infer using observed timeseries data of new cases, deaths and recoveries. In Bayesian inference these priors of the model parameters represent the initial beliefs about them which get updated (i.e., inferred) in the light of the incoming data. These prior values are extracted from previous studies which are given in the Table legend. We have copied these below for your convenience:

 “ **Secondary sources** (Huang et al., 2020; Kissler et al., 2020; Mizumoto and Chowell, 2020; Russell et al., 2020; Verity et al., 2020; Wang et al., 2020) and:

- https://www.statista.com/chart/21105/number-of-critical-care-beds-per-100000-inhabitants/
- https://www.gov.uk/guidance/coronavirus-COVID-19-information-for-the-public
- http://www.imperial.ac.uk/mrc-global-infectious-disease-analysis/COVID-19/

These prior expectations should be read as the effective rates and time constants as they manifest in a real-world setting. For example, a four-day period of contagion is shorter than the period that someone might be infectious (Wölfel et al., 2020) ^^[1]^^ , on the (prior) assumption that they will self-isolate, when they realise they could be contagious.”

- *How was the model fitted to data? Are you using MCMC? MLE? EM?*

The data fitting procedure is based on the standard variational (Bayesian) inference procedures (Beal, 2003) under Laplace approximation which is very similar in spirit to the EM algorithm. In our implementations we use Variational Laplace to evaluate model evidence *p*( *y*| *m*) and the posterior density over model parameters *p*( *θ*| *y*, *m*). For details of the procedures used, please see [1]  where we applied these procedures to the brain imaging timeseries or [2] for an application to the epidemiological timeseries data.

 The EM algorithm can be thought of as a simplified version of variational Bayes, in which the posterior distribution is approximated with the product of two marginals (e.g., over parameters and hyperparameters). The ensuing two variational updates correspond to the E and the M steps respectively. The simplification in the EM algorithm is that one assumes the approximate posterior is a point mass in the M-step.

- *The model does not include the influence of repeated seeding. For example, the UK has detected lots of introductions of SARS-CoV-2 cases, which is much higher than the assumed number of initial cases in this study. 1*

Dr Wang is absolutely right this model does not incorporate reseeding. We have now included the following (towards the end of the Conclusion section):

 “This DCM does not include the influence of repeated seeding. For example, the UK has detected introductions of SARS-CoV-2 cases, which is much higher than the assumed number of initial cases in this study (Firth et al., 2020). However, for simplicity, we have assumed that the community transmission had, quantitatively, a much greater contribution than reseeding. Having said this, it would be interesting to compare models with and without reseeding to test this assumption formally.”

- *The assumptions on contact patterns are oversimplified. Please account for age-stratified contact patterns.* *2*

We have now added the reference and our explanation in toward the end of the Conclusion section.

 “In this work, the model assumptions on contact patterns are simplified for e.g. age-stratified contact patterns are not considered (du Plessis et al., 2020). However, the current extensions of the model with eight stratification and contact matrices have been developed and are available in our open-source code base (DEM_COVID_S.m). Recent work on age stratified models using ONS data with age demographics now underwrites the dashboard ^8^ based upon the current DCM (DEM_COVID_UK.m). However, in the current model, we have simply lumped together all age groups—and have focused on heterogeneity in contact rates by conditioning them on different locations in the location factor.”

- *The authors assumed that self-isolated cases could not infect others. This is somehow problematic. For example, if a case was isolated at home, he/she can infect household members.*

We are operationally defined isolation as isolation from household members. Conversely, the location state ‘home’ allows for contacts with household members. In fact, the number of contacts at home is a free parameter of the model. This location state stands in for a low-contact risk state, in contrast to high contact risk states (e.g., grounded workplaces, public transport, football matches et cetera).

 References:

 [1] Friston KJ, Mattout J, Trujillo-Barreto N, Ashburner J, Penny W. Variational free energy and the Laplace approximation. Neuroimage. 2007 Jan 1;34(1):220-34. doi: 10.1016/j.neuroimage.2006.08.035. Epub 2006 Oct 20. PMID: 17055746.

 [2] Friston KJ, Parr T, Zeidman P *et al.* Dynamic causal modelling of COVID-19 [version 2; peer review: 2 approved]. *Wellcome Open Res* 2020, **5**:89 ( https://doi.org/10.12688/wellcomeopenres.15881.2)

# Reviewer report: Reviewer response for version 1

**DOI:** 10.21956/wellcomeopenres.17553.r39476

**Published:** 2020-08-05

**Authors:** Marta Colaneri

**Affiliations:** 1 Division of Infectious Diseases I, Fondazione IRCCS Policlinico San Matteo, Pavia, Italy

This paper presents a thorough analysis of the effects of FTTI (find, track, trace, isolate) in the dynamics of the pandemic in UK. The theoretical development follows one previous paper of the authors where a dynamic causal model of COVID-19 has been proposed. A new state (isolation state) is here added to model the effect of FTTI on the potential occurrence of the second wave of infection as a function of the rate at which immunity is lost in the first wave. A comparison between lethality in UK and Germany is discussed. 

 I believe that the paper might be useful for political decision-makers in phases 2 and 3.

 Nevertheless, I ask the authors to comment on the following issues, some of them criticizing the final conclusions: 

1. The underlying epidemiological model is quite complex with four macrostates-factors (Infection, Location, Testing, Symptoms), each subdivided in mutually exclusive states. As far as I can understand, the model is a mean-field approximation (to the first moments?) of an “atomic” model of the hiddel Markov chain, with equal individual propensities to change the state.In order to fully understand the temporal evolution (Kolmogorov forward bilinear equation)of the marginal probabilities of the 18 states it would be nice (if space allows) to have the mathematical model displayed along with the transition probability parameters.Does the model incorporate the probability distribution of the pairs in different factors?
2. In the legend of Fig. 1 it is written that one can move from the deceased state to the asymptomatic state, in order to preserve the probability mass (sum=1). Usually, the compartment of deaths is the integral of a linear combination of other states (and hence monotonically increasing). Could you please comment further?
3. There are indeed no absorbing states if immunity can be lost. However (as apparent from the simulations) an immunity time of 16 months seems to be large enough to drive the marginal probabilities of the infection status to constant values (“quasi” equilibrium). Could you please comment on the one-step transition probability from resistant to susceptible?
4. In standard epidemiological models (like SEIR)the effective reproduction number is the product between the basic reproduction number and the fraction of susceptible. Stability of the epidemic equilibrium can be characterized easily. I wonder how the theoretical development (of stability and convergence) can be done with time-varying (testing) parameters and Zeno-like behavior of the trajectories if other infection waves occur (in the absense of vaccine or effective therapies).
5. It seems that the resistant status in the infection factor is constant over time, i.e. its probability not affected by other status in other factors. Its value (0.38 in UK, 0.58 in DE) seems to be rather high and of course enforce herd immunity (low number of suceptibles left).
6. Could the model respond to the following question: who and where to test at observation time t+1 in order to maximize the detection of infected people? This question is motivated by the feeling that the second wave (small in terms of infections, and even before immunity loss) is the summation of very small infection clusters distributed in the territory, and a random network extension of an epidemiological models (endowed with node statistics ) is highly welcome.In the same vein, the “return infections” seems to be important after the international border disclosures.
7. Estimation of the number of infectives (in the “hidden” compartment) from noisy data (diagnosized infective, healed, deaths, number of tests etc…) is a real challenge and different approaches are on stage, with very different outcomes (number of total cases from 2 times to 15 times the number of diagnosized cases). How the results of the present paper are settled in this issue?, see e.g.the recent publications:

[*] Boehning at all, “Estimating the undetected infections in the Covid-19 outbreak by harnessing capture–recapture methods”, J. Infection Disease, 2020. ^1^

 [**] Philipps et al, “Estimating the true (population) infection rate for COVID-19: A Backcasting Approach with Monte Carlo Methods”, https://doi.org/10.1101/2020.05.12.20098889. ^2^

 I strongly agree with the sentence: this narrative depends on the acquisition of herd immunity …. However, in my view the inferred evidence of immunity in Figure 11 (Streek *et al*) is overestimate, being based on data collected in a super spread event. The difference of lethality with Germany is justified by the difference of resistant host of the population, and not on the greater propensity to test and tracing, availability of ICU beds and excellent functioning of the territorial health system, as claimed in a recent guest post by L. Wieler at al, “Emerging COVID-19 success story: Germany’s strong enabling environment”, June 30, 2020.

Is the rationale for developing the new method (or application) clearly explained?

Yes

Is the description of the method technically sound?

Partly

Are the conclusions about the method and its performance adequately supported by the findings presented in the article?

Yes

If any results are presented, are all the source data underlying the results available to ensure full reproducibility?

Yes

Are sufficient details provided to allow replication of the method development and its use by others?

Yes

Reviewer Expertise:

Infectious Diseases

I confirm that I have read this submission and believe that I have an appropriate level of expertise to confirm that it is of an acceptable scientific standard, however I have significant reservations, as outlined above.

## References

[1] : Estimating the undetected infections in the Covid-19 outbreak by harnessing capture-recapture methods. *Int J Infect Dis* .2020;97: 10.1016/j.ijid.2020.06.009 197-201 10.1016/j.ijid.2020.06.009 32534143 PMC7286831

[2] : Estimating the true (population) infection rate for COVID-19: A Backcasting Approach with Monte Carlo Methods. *medRxiv* .2020; 10.1101/2020.05.12.20098889 10.1101/2020.05.12.20098889

# Response

**Published:** 2021-02-04

**Authors:** Adeel Razi

**Affiliations:** Monash University, Australia

Dear Dr. Colaneri,

 We would like to thank you for the time and effort you have spent reviewing our manuscript. Below are the replies to the comments. We hope these revisions are what you had in mind.

- *The underlying epidemiological model is quite complex with four macrostates-factors (Infection, Location, Testing, Symptoms), each subdivided in mutually exclusive states. As far as I can understand, the model is a mean-field approximation (to the first moments?) of an “atomic” model of the hidden Markov chain, with equal individual propensities to change the state. In order to fully understand the temporal evolution (Kolmogorov forward bilinear equation) of the marginal probabilities of the 18 states it would be nice (if space allows) to have the mathematical model displayed along with the transition probability parameters. Does the model incorporate the probability distribution of the pairs in different factors?*

The transition probabilities between states within a factor can indeed depend on the current state in more than one factor. For example, the probability of dying after developing acute respiratory distress syndrome (ARDS) depends on whether one is in a critical care unit (CCU). The transition probabilities are outlined in detail in (Friston et al., 2020a) in the “Transition probabilities and priors” section. Updates for the present paper that incorporate new Infection and Location states are available in the code as described in the software note. We had anticipated that interested readers would refer to the first (foundational) paper in this series for the mathematical details (i.e., the master equation). However, we have added the following (second paragraph in section “Dynamic causal modelling”) who just want an intuition as to the basis of the model:

 “Although this model may look complicated; it is a straightforward compartmental model that can be written down in terms of a Master Equation, describing the discrete time updates of the joint probability distribution over the four factors. Please see (Friston et al., 2020a). Updating the joint probability (i.e., probability over all tuples of different states) allows us to model transitions among the states of one factor that depend upon other factors.”

- *In the legend of Fig. 1 it is written that one can move from the deceased state to the asymptomatic state, in order to preserve the probability mass (sum=1). Usually, the compartment of deaths is the integral of a linear combination of other states (and hence monotonically increasing). Could you please comment further?*

This was just for convenience. In other words, the dwell time in the deceased state can be read directly as the daily fatality rate (reported empirically). Transitions to the susceptible state assume that the number of people dying is balanced by the number of people being born, which is a simplifying but plausible approximation.

- *There are indeed no absorbing states if immunity can be lost. However (as apparent from the simulations) an immunity time of 16 months seems to be large enough to drive the marginal probabilities of the infection status to constant values (“quasi” equilibrium). Could you please comment on the one-step transition probability from resistant to susceptible?*

Although there are no absorbing states, having a very slow transition from the resistant state to the susceptible state can engender a quasi-equilibrium over short timescales. The slow transition from the resistant state to the susceptible state stands in for a loss of cross immunoreactivity, T cell memory – or indeed, an exchange of resistant individuals with another country (with predominantly susceptible individuals).

- *In standard epidemiological models (like SEIR)the effective reproduction number is the product between the basic reproduction number and the fraction of susceptible. Stability of the epidemic equilibrium can be characterized easily. I wonder how the theoretical development (of stability and convergence) can be done with time-varying (testing) parameters and Zeno-like behavior of the trajectories if other infection waves occur (in the absence of vaccine or effective therapies).*

We agree that the stability of and convergence to equilibrium points – under different testing scenarios — are of great interest, and have begun to consider this question in a subsequent manuscript which is currently under peer review: https://www.medrxiv.org/content/10.1101/2020.06.11.20128611v1

- *It seems that the resistant status in the infection factor is constant over time, i.e. its probability not affected by other status in other factors. Its value (0.38 in UK, 0.58 in DE) seems to be rather high and of course enforce herd immunity (low number of susceptible left).*

The resistant proportion is estimated from the data and these values, which are by definition higher than those in models that assume that 100% population susceptibility to a novel virus, offer the best account for the data under the current model structure. In subsequent iterations of the model (one published at https://wellcomeopenresearch.org/articles/5-204/v2 and another under peer review https://www.medrxiv.org/content/10.1101/2020.09.01.20185876v1), we separately consider heterogeneity of exposure, susceptibility, and transmission. This allows for the possibility that a subset of those we define here as “resistant” may still pick up the virus, test positive in PCR tests, but not (so readily) transmit the virus to others. We are also developing the model to allow for changes in the exposed population, for example through population fluxes ( https://www.fil.ion.ucl.ac.uk/spm/covid-19/TR5_Second_Wave.pdf and https://www.fil.ion.ucl.ac.uk/spm/covid-19/TR6_DCM.pdf), but this is probably beyond the scope of the current report.

- *Could the model respond to the following question: who and where to test at observation time t+1 in order to maximize the detection of infected people? This question is motivated by the feeling that the second wave (small in terms of infections, and even before immunity loss) is the summation of very small infection clusters distributed in the territory, and a random network extension of an epidemiological models (endowed with node statistics ) is highly welcome. In the same vein, the “return infections” seems to be important after the international border disclosures.*

Local versions of the model at the level of Local Authorities in England have been implemented, the results of which are viewable at https://fil.ion.ucl.ac.uk/spm/covid-19/dashboard/local/ alongside actual test result data. This kind of dashboard could in principle be used to guide decisions on where to focus testing resources to maximize detection of infection. We do not currently model connections between these regions, although we are considering in ongoing work the relative impact of population fluxes (“return infections”) versus loss of individual immunity on the development of secondary and second waves of infection.

- *Estimation of the number of infectives (in the “hidden” compartment) from noisy data (diagnosed infective, healed, deaths, number of tests etc.…) is a real challenge and different approaches are on stage, with very different outcomes (number of total cases from 2 times to 15 times the number of diagnosed cases). How are the results of the present paper settled in this issue? See e.g., the recent publications:*

          [*] Boehning et al, “Estimating the undetected infections in the Covid-19 outbreak by harnessing              capture–recapture methods”, J. Infection Disease, 2020.

[**] Philipps et al, “Estimating the true (population) infection rate for COVID-19: A Backcasting                 Approach with Monte Carlo Methods”

 We have now included the references provided by the reviewer and added (in the Conclusion)

 “Here we also note that the estimation of the true number of infected people from noisy timeseries data is a real challenge and different approaches are proposed, with very different outcomes (number of total cases from 2 times to 15 times the number of diagnosed cases), see (Bohning, Rocchetti, Maruotti, & Holling, 2020; Phipps, Grafton, & Kompas, 2020). It is indeed the potential to estimate the true number of infected people (and other key latent variables) that led us to adopt and advocate the generative modelling approach of DCM, coupled with variational Bayesian inference that allows us to quantify and account for uncertainty in the case and death data. A validation of these kinds of estimates, with those provided by Edge Health, was recently reported in the Guardian .”

- *I strongly agree with the sentence: this narrative depends on the acquisition of herd immunity …. However, in my view the inferred evidence of immunity in Figure 11 (Streek et al) is overestimate, being based on data collected in a super spread event.*

We acknowledge that seroprevalence varies considerably with the nature of the population and events under consideration. Furthermore, seropositive prevalence may not provide the complete picture, especially in the setting of heterogeneity of susceptibility to infection. We have pursued this in subsequent papers testing the notion of seronegative resistance as a potential explanation for differences between countries – much along the lines suggested by Dr. Colaneri.

  

- *The difference of lethality with Germany is justified by the difference of resistant host of the population, and not on the greater propensity to test and tracing, availability of ICU beds and excellent functioning of the territorial health system, as claimed in a recent guest post by L. Wieler at al, “Emerging COVID-19 success story: Germany’s strong enabling environment”, June 30, 2020.*

We agree entirely with Dr. Colaneri’s observations, which were borne out in subsequent applications of this model to the unfolding trajectories in several European countries.

- *Further note: Y-axes in Fig. 3 are incorrect, should be something like “reproduction rate” and “proportion (%)”*

Thank you for pointing this out. These typos are now corrected.
